# A Regional Initiative to Reduce Skin Infections amongst Aboriginal Children Living in Remote Communities of the Northern Territory, Australia

**DOI:** 10.1371/journal.pntd.0000554

**Published:** 2009-11-24

**Authors:** Ross M. Andrews, Therese Kearns, Christine Connors, Colin Parker, Kylie Carville, Bart J. Currie, Jonathan R. Carapetis

**Affiliations:** 1 Menzies School of Health Research, Charles Darwin University, Darwin, Northern Territory, Australia; 2 Northern Territory Department of Health and Families, Darwin, Northern Territory, Australia; 3 Australasian College of Dermatologists, Boronia Park, New South Wales, Australia; 4 Murdoch Childrens Research Institute, Melbourne, Victoria, Australia; Massachusetts General Hospital, United States of America

## Abstract

**Background:**

Linked to extreme rates of chronic heart and kidney disease, pyoderma is endemic amongst Aboriginal children in Australia's Northern Territory (NT). Many of those with pyoderma will also have scabies. We report the results of a community-based collaboration within the East Arnhem Region, which aimed to reduce the prevalence of both skin infections in Aboriginal children.

**Methodology/Principal Findings:**

Commencing September 2004, we conducted an ecological study that included active surveillance for skin infections amongst children aged <15 years in five remote East Arnhem communities over a three year period. Screening was undertaken by trained local community workers, usually accompanied by another project team member, using a standard data collection form. Skin infections were diagnosed clinically with the aid of a pictorial flip chart developed for the purpose. Topical 5% permethrin was provided for age-eligible children and all household contacts whenever scabies was diagnosed, whilst those with pyoderma were referred to the clinic for treatment in accordance with current guidelines. In addition, annual mass scabies treatment (5% permethrin cream) was offered to all community residents in accordance with current guidelines but was not directly observed. Pyoderma and scabies prevalence per month was determined from 6038 skin assessments conducted on 2329 children. Pyoderma prevalence dropped from 46.7% at baseline to a median of 32.4% (IQR 28.9%–41.0%) during the follow-up period – an absolute reduction of 14.7% (IQR 4.7%–16.8%). Compared to the first 18 months of observation, there was an absolute reduction in pyoderma prevalence of 18 cases per 100 children (95%CI −21.0, −16.1, p≤0.001) over the last 18 months. Treatment uptake increased over the same period (absolute difference 13.4%, 95%CI 3.3, 23.6). While scabies prevalence was unchanged, the prevalence of infected scabies (that is with superimposed pyoderma) decreased from 3.7% (95%CI 2.4, 4.9) to 1.5% (95%CI 0.7, 2.2), a relative reduction of 59%.

**Conclusion:**

Although pyoderma prevalence remained unacceptably high, there was a substantial reduction overall with improvements in treatment uptake a critical factor. More acceptable alternatives, such as cotrimoxazole for pyoderma and ivermectin as a community-wide scabicide, warrant further investigation in these settings. We are encouraged by progress made through this work, where local action was led by local community members and primary health care providers with external training and support.

**Trial Registration:**

ClinicalTrials.gov NCT00884728

## Author Summary

Skin infections are endemic in many in remote Australian Aboriginal communities and have been linked to very high rates of chronic heart and kidney disease in this population. We report the results of a regional collaboration that aimed to reduce skin infections amongst children aged less than 15 years in five remote communities. The program included annual mass scabies treatment days offered to all residents and routine screening/follow-up of children. Trained community workers helped conduct over 6000 skin assessments on 2329 children over a three year period. Of every 100 children seen at the commencement of the study, 47 were found to have skin sores and many had multiple sores. We demonstrate a reduction both in the number of children with skin sores and in the severity of those sores. On average, of every 100 children seen per month, there were 14 fewer children with skin sores and seven fewer children with multiple sores. Overall improvement in treatment uptake was a critical factor. We found no discernible impact against scabies. While the burden of skin infections remains unacceptably high, we believe the results presented here are a good news story for local action to address a serious public health problem.

## Introduction

Pyoderma is a generic term used to describe a clinical diagnosis of superficial bacterial skin infection [1]. Also known as skin sores or impetigo, it has been estimated that there are in excess of 111 million children with pyoderma worldwide and that many of these children will also have scabies [1]. Reported pyoderma prevalence has varied, but children living in Australian Aboriginal communities and those living in the Pacific region have generally had the highest burden, often in the range of 40–90% [2]. A recent study from Fiji [3], reported pyoderma prevalence of 25% amongst primary school children and 12% amongst infants. Like previous studies in remote Australian Aboriginal communities [4],[5], the Fijian study found that children with scabies were more likely to have pyoderma and that most pyoderma was due to Group A streptococcal (GAS) infection. Multiple GAS strains can be present at the same time in remote Australian Aboriginal communities, with household acquisition rates as high as 1 in 5 [6]. Whereas one or two sores would raise alarm in other settings, the burden amongst Aboriginal children is such that current recommendations for the Northern Territory (NT) specify “six infected sores” as one of the benchmarks for pyoderma treatment [7].

Controlling pyoderma and scabies could be an important primary health intervention to reduce serious bacterial infections in childhood and may have potential longer term sequelae, particularly in relation to acute rheumatic fever (ARF), rheumatic heart disease (RHD) and chronic kidney disease. Pyoderma is a major underlying cause of serious bacterial infections (GAS and *Staphylococcus aureus*), which are very common in Aboriginal communities and have high mortality [8],[9]. The burden of ARF/RHD is extremely high amongst Indigenous Australians [10]. Since GAS throat infection is rare in this setting, it seems probable that pyoderma (and scabies) may have a key role in the ARF/RHD burden, although the causal pathway remains uncertain [9],[11],[12]. Over the past decade: the Indigenous incidence of end stage renal disease has been shown to be 21 times that of non-Indigenous Australians [13]; the risk of acute post streptococcal glomerulonephritis (APSGN) five times higher for children with pyoderma during APSGN outbreaks [14],[15]; and APSGN in childhood has been shown to substantially increase the risk of adult renal disease [16]. Mortality from kidney failure is also much higher than non-Indigenous Australians [17].

Menzies School of Health Research and others have collaborated with NT communities and the government authorities over more than 10 years to develop strategies for tackling skin infections at a community level. The initial work, which documented the link with severe and chronic diseases, included laboratory based studies to demonstrate that dog and human scabies were not linked [18] and that the diverse range of streptococcal strains – including those associated with rheumatic fever and kidney disease – are all derived from skin infections [19]. A successful community based scabies control program, involving annual mass treatment days for scabies (using topical 5% permethrin cream) and active surveillance for skin infections, was conducted in one of the largest remote communities [20]. A feasibility study subsequently demonstrated widespread support for similar programs throughout NT Indigenous communities noting those communities that had successfully implemented their own programs were keen to participate in coordinated programs with related communities [21]. We report the results of a regional collaboration involving a number of these communities in the East Arnhem Region of the NT where the primary aim was to reduce the prevalence of scabies and pyoderma amongst children aged 0–14 years.

## Methods

### Setting and Population

The East Arnhem Healthy Skin Project (EAHSP) involved five remote communities and associated homelands/outstations. The total population of each community ranged from approximately 800–2000 people, encompassing a total of approximately 2600 Indigenous children aged <15 years. Active surveillance for skin infections was conducted over a three year period from September 2004–August 2007. Children aged <15 years were screened for skin infections at either the local health centre, home, in the community or at school using a standard data collection form. We collected additional data for a subset of children seen during school screening, which included: appearance of pyoderma (crusted, purulent or flat/dry), site of pyoderma (upper body or lower body), the number of sores (<5, 5–20 or >20) and, for those with scabies, whether or not the infestation had become infected (scabies with a superficial bacterial skin infection  =  pyoderma [1]). Screening was undertaken by trained local community workers, usually accompanied by another project team member (Aboriginal Health Worker, registered nurse, or visiting pediatrician/dermatologist).

A formal training program was established for community workers involving both practical “on-the-job” training and formal learning packages delivered as “off-the-job” components. Eleven workers completed the training program and gained recognition against units of competency towards a Primary Health Care qualification. The community workers, all local Aboriginal people, were employed for up to 20 hours per week to undertake skin screening of children and were supported every 1–2 months by a visiting research team member.

Annual healthy skin days were held at each community, commencing from September 2004. Coordinated by the local community, these events included promotion of mass treatment with scabicide cream (distributed in 30g tubes with 5% permethrin, *Lyclear* ®) for all community members in accordance with current guidelines [22]. The cream was hand delivered to each household with adequate quantities supplied for all household members and verbal advice provided on appropriate use of the cream. Treatment application was not directly observed as this was not considered culturally appropriate.

### Ethics Statement

The project was approved by the Human Research Ethics Committee of the Northern Territory Department of Health Community Services and Menzies School of Health Research (Approval number 04/11) with written informed consent obtained for participants.

### Screening and Diagnosis

We developed two pictorial flipcharts, one to explain the Healthy Skin Story in lay terms for study participants which included family-based and environmental activities [23], the other to assist in diagnosis focusing primarily on recognition and treatment of pyoderma, scabies and tinea [24]. Both flip charts were used by all study staff and distributed to each participating community. Skin infections were diagnosed clinically by the research team. Pyoderma lesions were not routinely swabbed nor were scabies mites routinely extracted.

### Treatment

Children with pyoderma were referred to the clinic for treatment in accordance with the current guidelines [7]. If children screened by the research team were identified with scabies, the parent/carer was given topical 5% permethrin (*Lyclear* ®) to administer to the child and all household contacts. For infants younger than 2 months of age topical Crotamiton 10% cream was used.

### Objectives and Outcomes

Given previous experience of Healthy Skin programs [4],[5],[20], we expected to observe a reduction in both scabies and pyoderma prevalence immediately following the initial mass scabicide treatment. Our primary objective was to demonstrate reductions in both conditions among children aged 0–14 years in the participating communities within two years following the introduction of the program:

- For scabies, we aimed to reduced prevalence from 30% (expected pre-program) to <10%.
- For pyoderma, we aimed to reduce prevalence from 50% (expected pre-program) to <25%.

A secondary objective was to reduce the severity of pyoderma among the target group that was classified as moderate/severe from 40% (expected pre-program) to <15%.

### Statistical Analyses

We determined the prevalence of pyoderma and scabies at baseline as the proportion of all children seen in September 2004 (immediately preceding the initial mass community-wide scabicide treatment). Thereafter, we determined the monthly period prevalence of both pyoderma and scabies on the same basis, calculated as a rolling average of all children seen over the preceding three-month period (ie commencing from December 2004 through to August 2007). Although some children were seen more frequently, we excluded subsequent assessments that occurred within 30 days of the initial assessment in order to ensure that only one assessment per individual per month was included.

These were population level surveillance data (non-random sample) where the intention was to screen as many children as possible within the region given the available resources. We monitored variation in the number of children seen over the surveillance period by determining the median number, and interquartile range (IQR), of children seen over each of the rolling three-month periods. In addition, we were cognizant of the potential that eagerness to demonstrate a benefit from a Healthy Skin program might preferentially encourage screening of healthier children or that awareness of the program might conversely result in an increase in care-seeking behaviour for those children with skin infections. We monitored this potential by conducting an exploratory sub-analysis of pyoderma and scabies prevalence on the first occasion that an individual subject was seen.

We examined seasonal differences by comparing the average monthly prevalence during the wet season (September–February) and the dry season (March–August). We also monitored changes over time by comparing the average monthly prevalence during the first 18 months of the study period to that for the last 18 months using the 2 sample test of proportions as an exploratory analysis. Data were analysed using Stata version 9 software.

## Results

### Number of Screening Assessments

**Figure 1:**
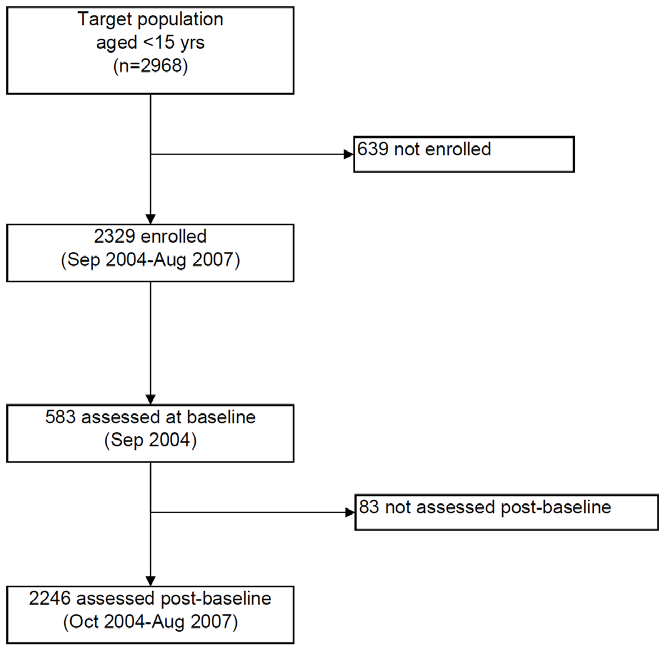
Participation in EAHSP, September 2004–August 2007.

There were 6038 skin assessments conducted on 2329 children during the three year study period, estimated to represent 78% of the target population (Figure 1). Most children (1245, 53.5%) were seen once or twice, 19% three times and 28% were seen from four to nine times. There were 583 children screened during the initial assessment in September 2004 with a similar number (median 487, IQR 405–578) screened over the subsequent rolling three-month periods to August 2007 (Table 1). The median number of children seen for the first time during the post baseline period was 132 (IQR 92–211), that is approximately 1 in 4 children seen during the rolling 3 month periods after September 2004 had not previously been seen. Overall, there were 2001 assessments performed by study team members at school (33%) and 4037 (67%) elsewhere within the community (eg home or clinic).

**Table 1:** Population-based screening for pyoderma and scabies among children aged <15 years, East Arnhem Region, September 2004–August 2007.

		Pyoderma		Scabies	
		n	% (95% CI)	n	% (95% CI)
Children screened for skin condition^a^		2323	n/a	2321	n/a
Average monthly prevalence all ages^b^		6018	35.5% (34.3,36.8)	6026	13.4% (12.5,14.3)
	Aged <3 yrs	1192	35.6% (32.9,38.3)	1198	22.7% (20.3,25.1)
	Aged 3-<9 yrs	2925	39.8% (38.1,41.6)	2925	13.3% (12.1,14.6)
	Aged 9–14 yrs incl	1901	28.9% (26.9,31.0)	1903	7.6%, (6.4,8.8)
Median monthly prevalence		**n**	**% (IQR)**	**%**	**% (IQR)**
September 2004 (Baseline)		582	45.7%	583	16.1%
October 2004–August 2007 (all assessments)^c^		487	32.4% (28.9–41.0)	487	13.1% (11.7–15.9)
October 2004–August 2007 (1^st^ assessment)^d^		132	34.5% (29.3–43.4)	132	15.8% (12.9–19.4)

a “n” refers to the number of children seen. In total, there were 2329 children for whom skin assessments undertaken, 6 children (0.3%) had data missing on pyoderma status and 8 children (0.3%) had data missing on scabies status.

b “n” refers to the number of skin assessments undertaken. In total, there were 6038 skin assessments undertaken on 2329 children with 1084 children seen more than once (range 2–9 times). Twenty assessments (0.3%) had data missing on pyoderma status, and 12 (0.2%) on scabies status.

c “n” refers to the median number of children screened during rolling 3 month periods from October 2004–August 2007 (ie 33 consecutive periods). The interquartile range was 405–578 for pyoderma and 405–580 for scabies.

d “n” refers to the median number of children screened for the first time during rolling 3 month periods from October 2004–August 2007 (ie 33 consecutive periods). The interquartile range for the first assessment was 92–211 for pyoderma and for scabies.

### Pyoderma and Scabies

By August 2004, there had been 2139 episodes of pyoderma diagnosed and 807 episodes of scabies. Of the 2329 children seen, almost all (91.8%, 95%CI 87.7,96.0) had pyoderma on at least one occasion, while 34.7% (95%CI 31.9,37.4) had scabies on at least one occasion. The average monthly prevalence was 35.5% for pyoderma and 13.4% for scabies (Table 1). Pyoderma prevalence was slightly higher amongst males, 38.8% (95%CI:37.1,40.5), than females, 32.1% (95%CI:30.4,33.8) – an absolute difference of 6.7% (95%CI:4.3,9.1). There was no evidence of a seasonal difference in pyoderma prevalence: wet seasons = 35.3% (95%CI:33.5,37.0); dry seasons = 35.8% (95%CI:34.1,37.5).

Pyoderma prevalence was similar between the three age groups. In contrast, scabies prevalence was highest amongst children aged <3 years and decreased with age (Table 1). Scabies prevalence amongst children aged <3 years (22.7%, 95% CI 20.3, 25.1) was double that of children aged 3–14 years (11.1%, 95% CI 10.2, 12.0).

Whereas pyoderma prevalence at baseline (46.7%) was close to expectations, scabies prevalence at baseline (16.1%) was substantially lower than we expected (30%). Thereafter, we observed a reduction in pyoderma prevalence but little if any change in scabies prevalence. During the post baseline period, the median monthly prevalence of pyoderma was 32.4% (IQR 28.9–41.0%) – an absolute reduction of 14.7% (IQR 4.7–16.8%). A similar reduction was evident when prevalence was limited to the first time an individual child was seen – absolute reduction of 11.2% (IQR 2.3–16.4%). In contrast, the median monthly prevalence of scabies remained similar at 13.1% (IQR 11.7–15.9) – a marginal absolute reduction of 3.0% (IQR 0.2–4.4%), with no discernible difference in the median monthly prevalence when limited to children seen for the first time during the post baseline period (Table 1).

**Figure 2:**
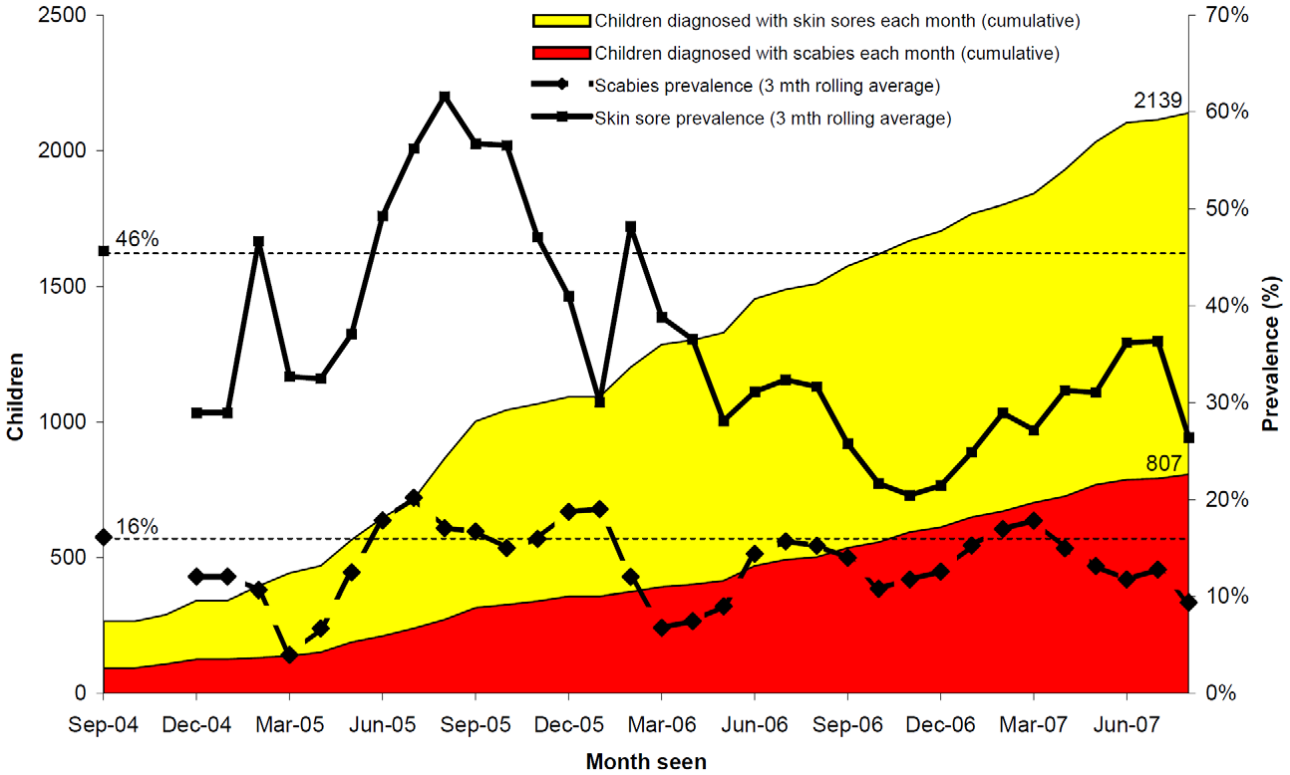
Pyoderma and scabies among children aged <15 years. n = 6038 assessments conducted through community based surveillance for the East Arnhem Healthy Skin Project from September 2004–August 2007.

**Figure 3:**
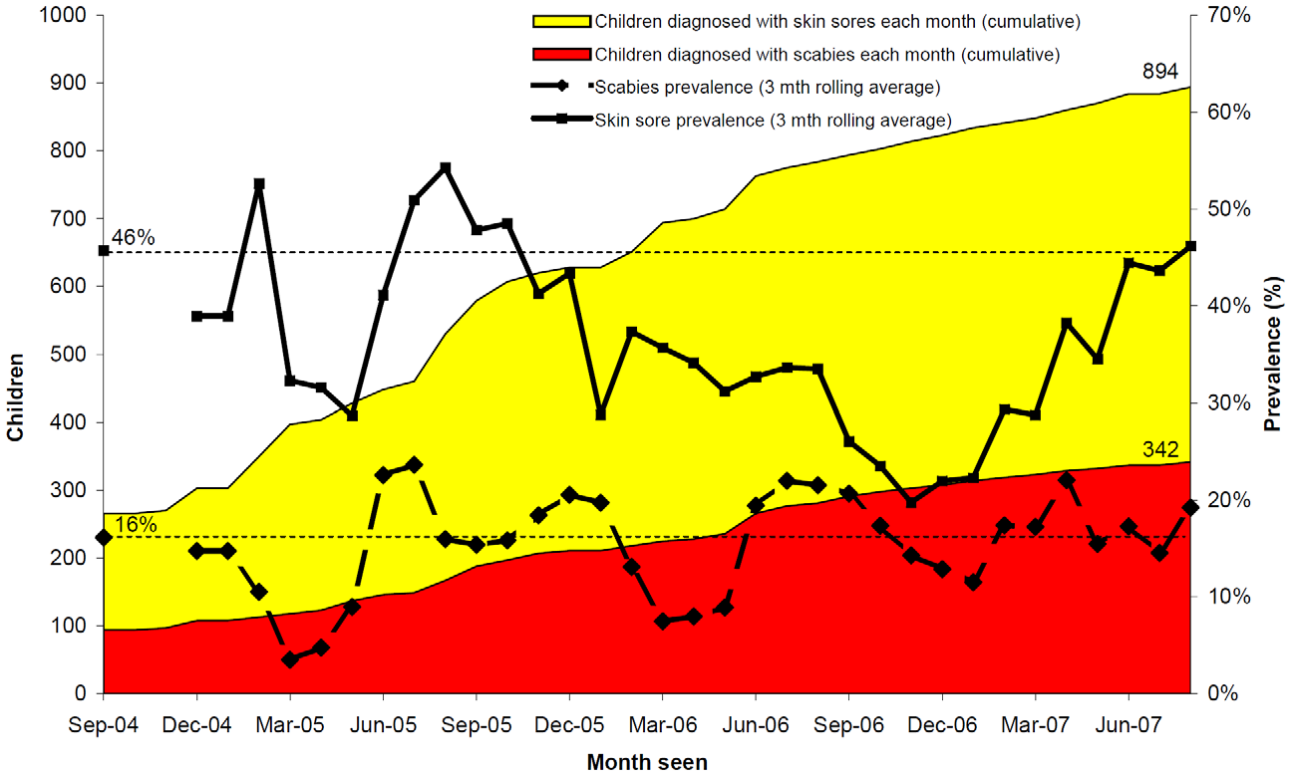
Pyoderma and scabies at the time of first assessment among children aged <15 years. n = 2323 assessments conducted through community based surveillance for the East Arnhem Healthy Skin Project from September 2004–August 2007.

Both pyoderma and scabies prevalence varied over time (Figure 2) but these did not closely correspond to annual mass scabicide treatment, which occurred in September 2004, September 2005 and September–October 2006. Whereas scabies prevalence fluctuated close to the baseline rate over the three year study period, pyoderma prevalence was notably lower over the latter 18 month period (Figure 2). For scabies, the pattern was similar when data were limited to prevalence determined amongst those children who were seen for the first time (Figure 3). In contrast, pyoderma prevalence amongst this subgroup of children actually increased towards the end of the observation period suggesting that these children were more likely to be diagnosed with pyoderma but were not more likely to be diagnosed with scabies.

**Figure 4:**
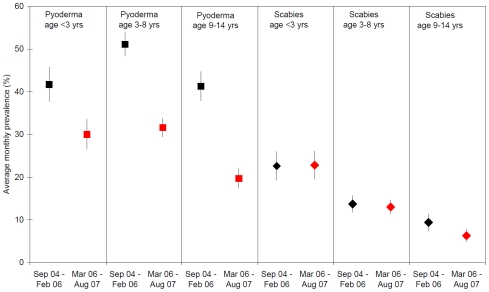
Average monthly prevalence of pyoderma and scabies by age group and month of screening. n = 6038 assessments conducted through community based surveillance for the East Arnhem Healthy Skin Project from September 2004–August 2007.

The reduction in pyoderma prevalence over the latter 18 month period was evident across all age groups (Figure 4), whereas there was no evidence of a comparable reduction in scabies prevalence. During the latter 18 month period, there were 18 fewer pyoderma cases for every 100 children seen (95%CI:−21.0,−16.1, p<0.001), a 40% reduction when compared to rates during the first 18 month period. The greatest absolute reduction was amongst children aged 3–14 years: 20 fewer cases of pyoderma for every 100 children seen (−20.3%, 95%CI:−23.0,−17.6).

We collected additional data for children seen during school screening (n = 2001 aged 3–14 years). For this subset of children, pyoderma prevalence was 40.2% (95%CI 38.1,42.4), slightly higher than that of children in the same age group who were seen outside the school setting (33.2%, 95%CI 31.8,34.7). For children seen during school screening, pyoderma was most commonly diagnosed on the legs (Table 2) where 36.5% of children had lower body pyoderma compared with 17.6% upper body pyoderma. Multiple sores (five or more) were also more common on the lower body (14.2%) than the upper body (4.5%). Of those school children with pyoderma, 88.1% (95%CI: 85.7,90.4) had sores on the lower body while 42.4% (95%CI:38.9,45.9) had sores on the upper body. The lower body was the region where the reduction in prevalence was most evident: 13 fewer cases of lower body pyoderma per 100 children seen and 6 fewer cases of children with multiple lower body sores (Table 2). There was also a reduction in the nature of the sores, with 10 fewer cases of crusted/purulent pyoderma per 100 children seen during the latter 18 month period.

**Table 2:** Average monthly prevalence of pyoderma and scabies, EAHSP, September 2004–August 2007.

	Sep04–Feb06 (18 months) % (95%CI)	Mar06–Aug07 (18 months) % (95%CI)	Absolute difference % (95%CI)	p
**Pyoderma**				
**All ages**	**46.0 (44.1,48.0)**	**27.5 (26.0,29.0)**	**−18.5 (−21.0,−16.1)**	**<0.001**
Aged <3 yrs	41.7 (37.7,45.8)	30.0 (26.4,33.6)	−11.8 (−17.2,−6.3)	<0.001
Aged 3-<9 yrs	51.1 (48.4,53.9)	31.6 (29.4,33.8)	−19.5 (−23.1,−16.0)	<0.001
Aged 9–14 yrs incl	41.3 (37.9,44.7)	19.7 (17.4,22.1)	−21.6 (−25.7,−17.5)	<0.001
***Additional data*** *				
*Upper body pyoderma* *	22.7 (19.8,25.6)	13.5 (11.4,15.6)	−9.2 (−12.8,−5.7)	<0.001
*Lower body pyoderma* *	44.1 (40.7,47.5)	30.4 (27.6,33.2)	−13.7 (−18.1,−9.3)	<0.001
*Upper body 5 or more sores*	5.2 (3.6,6.7)	4.0 (2.8,5.2)	−1.2 (−3.2,0.7)	0.2
*Lower body 5 or more sores*	17.8 (15.1,20.5)	11.5 (9.5,13.5)	6.3 (−9.6,−2.9)	<0.001
*5 or more sores* *	19.9 (17.2,22.7)	12.6 (10.6,14.7)	−7.3 (−3.9,−10.8)	<0.001
*Treatment for those with 5+ sores* *	3.8 (−0.4, 7.9)	17.2 (8.0,26.4)	13.4 (3.3,23.6)	0.007
*Crusted/purulent pyoderma* *	31.6 (28.4,35.8)	21.6 (19.0,24.1)	−10.0 (−14.1,−6.0)	<0.001
*Treatment for crusted/purulent pyoderma* *	3.9 (1.5,6.2)	7.3 (3.9,10.8)	3.5 (−0.7,7.6)	0.1
**Scabies**				
**All ages**	**14.3 (13.0,15.7)**	**12.7 (11.6,13.8)**	**−1.7 (−3.4,0.1)**	**0.06**
Aged <3 yrs	22.6 (19.2,26.0)	22.8 (19.5,26.1)	0.2 (−4.5,5.0)	0.9
Aged 3-<9 yrs	13.7 (11.8,15.7)	13.0 (11.4,14.6)	−0.7 (−3.2,1.8)	0.6
Aged 9–14 yrs incl	9.4 (7.4,11.4)	6.3 (4.9,7.8)	−3.1 (−5.5,−0.6)	0.01
***Additional data*** *				
Infected scabies*	3.7 (2.4,4.9)	1.5 (0.7,2.2)	−2.2 (−3.7,−0.7)	0.003

Notes: “Absolute difference” refers to the difference in the average monthly prevalence in the first 18 months (Sep04–Feb06) compared to the second 18 months (Mar06–Aug07).

*: indicates parameters for which comparable data were sought on 2001 children seen during school screening (age 3–14 years).

From the additional data collected, we identified 144 children who had been diagnosed with five or more pyoderma lesions. Of these, only 50 (34.7%) were recommended for treatment – the vast bulk, 94 (65.3%) had no treatment follow-up. We reviewed clinic records for those who had been referred for treatment. While treatment uptake remained low, there was a substantial improvement during the latter 18 month period when 17.2% of children with five or more sores received treatment, single dose benzathine penicillin, compared to 3.8% during the initial 18 month period (Table 2). For those with five or more sores, this was an absolute increase in treatment uptake equivalent to 13 children per 100 seen (95%CI 3.3,23.6). By comparison, although there was an overall reduction in crusted or purulent pyoderma (10%) there was not a demonstrable increase in treatment uptake for these children (3.5%, 95%CI −0.7,7.6).

While there was no reduction in scabies prevalence over the latter 18 month study period (Figure 4), there was a significant reduction in prevalence of infected scabies amongst children aged 3–14 years, falling from 3.7% (95% CI 2.4,4.9) of children seen in the first 18 months to 1.5% (95% CI 0.7,2.2) in the last 18 months (Table 2). Comparable data were not routinely collected over the entire study period for children aged less than 3 years.

## Discussion

Our data suggests regular surveillance and routine service delivery can have an impact on pyoderma prevalence, independent of a reduction in scabies prevalence, and that the reduction in pyoderma prevalence is probably due to increased adherence to antibiotic treatment regimens. This study was a collaboration between local people in remote Aboriginal communities of northern Australia, staff at the primary health care clinic, researchers and other health specialists. The concept for a regional approach was built on a history of more than a decade of work aimed at tackling skin infections at the community level [4],[20]. Our expectation was that mass community treatment for scabies would provide the impetus to reduce skin infections. This had been the experience previously, where reductions in scabies prevalence had been accompanied by reductions in pyoderma prevalence, predominantly of the upper body [4],[5]. In contrast, we found no evidence of an overall impact on scabies prevalence but a substantial reduction in pyoderma prevalence, predominantly (but not limited to) the lower body. These data were supported by evidence of increased treatment uptake for children with five or more sores and a trend towards increased treatment uptake for those with crusted or purulent sores.

While the data need to be considered with caution (as discussed later), it is encouraging to note that there was a reduction in the observed pyoderma prevalence over the study period. Compared to the initial screening in September 2004, the median prevalence in the post baseline period was equivalent to 14 fewer cases of pyoderma per 100 children seen (IQR 4.7–16.8). When the baseline period was extended to include the first 18 months of observation (September 2004–February 2006), the reduction over the following 18 month period was equivalent to 18 fewer cases of pyoderma per 100 children seen (95%CI 16,21). The reduction was observed across all age groups and, where data were available, was also seen across both lower and upper body sites, there were fewer children with multiple sores and fewer children with crusted/purulent pyoderma. We saw no overall reduction in scabies prevalence but there was a reduction in the proportion with infected scabies. Overall, these data are most consistent with a pyoderma treatment effect than an underlying reduction in scabies prevalence.

We have been encouraged by a model of outreach service that evolved during the study incorporating locally trained community workers at the front-line, well supported by local health service providers and community leaders. The increased surveillance of skin infections that was provided by the community workers, which included home visits, was beyond the current capacity/usual practice of local health-care clinics. The subsequent decline in pyoderma highlights the benefits of such a service for both community members and health services given the high workload generated by skin infections.

Although we are encouraged by the apparent decrease, the pyoderma burden remains unacceptably high, affecting in excess of 25 children out of every 100 seen each month for the last year of the study. In related work undertaken in two of the participating communities, we found that 50% of children presented to a primary health care clinic between 10 and 22 times per child per year and that almost 1 in 5 of these presentations (18%) were for skin infections [25]. Most children reached their first birthday having had an episode of pyoderma (69%) and scabies (63%), with most having their first episode diagnosed at just 2 months of age [25]. Previous work in a similar setting has shown secondary transmission of GAS within the household to be as high as 19% [6].

Pyoderma prevalence has ranged from 10–70% in other studies [4],[5]. Wong et al administered intramuscular benzathine penicillin at the initial diagnosis and again at follow up visits [20]; non-scabies pyoderma reduced from 11% to 3% within three months but returned to pre-intervention rates by nine months. In our study, pyoderma treatment was by referral to the primary health care clinic. We saw no evidence of a reduction during the initial 18 month period then observed a sustained reduction in pyoderma prevalence over the ensuing 18 month period.

Given the high endemicity of pyoderma in remote Indigenous communities, there may well be a tendency for multiple sores to be seen as the norm rather than being a serious condition requiring treatment. This seems to be reflected in current guidelines for the area that recommend treatment where there are “six infected sores” or the sores “look severe” [7]. In our view, the threshold for pyoderma treatment has been raised too high. Our data support this concern. Of 1818 children from whom data were recorded on the number of sores, we found 1 in every 6 children screened (16%) had multiple sores but (even though treatment uptake improved) very few ultimately received that treatment. Clearly, appropriate treatment of children with severe pyoderma is an area with substantial scope for improvement. Overall, treatment uptake remained sub-optimal but we were encouraged by the improvement in pyoderma treatment rates during the latter 18 month period and suspect that this may have contributed to the reduction in prevalence. Low treatment uptake rates and the emerging issue of community-associated methicillin-resistant *Staphylococcus aureus* in Indigenous populations [26], highlight the need to investigate alternatives to benzathine penicillin for pyoderma treatment.

Scabies prevalence in our study was lower than previously published data [4], but still substantial - particularly for younger children where the monthly period prevalence was 23% for those aged <3 years. In contrast to other settings where community-wide use of scabicides has reduced scabies prevalence [5],[20],[27], it is apparent that household scabicide use within our communities has been poor. In a nested study, La Vincente et al found only 44% of household contacts of scabies cases used the permethrin cream [28]. Given that those households where all contacts had used the cream were six times less likely to have ongoing scabies transmission [28], low levels of household treatment uptake appears the most likely explanation for the lack of a discernible impact against scabies prevalence. We acknowledge that permethrin resistance is of concern [29], however the findings of the nested study by La Vincente et al suggest the issue is primarily related to non-use of the cream rather than a failure of cream itself. Alternative treatment regimes, in particular the role of oral ivermectin in older children and household contacts [30],[31], warrants further investigation in this setting. Licensed to treat onchocerciasis and strongyloidiasis, ivermectin has also been approved for treatment of refractory crusted scabies cases in Australia but is not currently approved for treatment of ‘ordinary’ scabies cases and their contacts [32].

Determining the denominator for a prospective study such as ours is problematic as there can be considerable population movement between communities over time. We also acknowledge the potential bias in care-seeking/screening seeking behaviour and also the lack of control for potential confounding factors. However, given that scabies and pyoderma are so frequently identified as co-infections in children, it would seem unlikely that bias or confounding could explain why prevalence of the former would be unchanged while the latter reduced substantially.

### Conclusions

Sub standard living conditions are common factors reported in many communities where skin infections are endemic [33],[34]. Redressing inadequate living conditions, poverty and other social determinants of health are major priorities for long term health benefits for Indigenous Australians. For skin infections, a reduction in pyoderma prevalence is the primary priority and the most encouraging outcome from our study. In our view, increased treatment uptake is the most likely explanation but we believe it has been local action delivered through basic primary health services that has been the driver.

Surveillance for skin infections, supported by appropriate treatment where indicated, can reduce pyoderma prevalence. Local community workers played a key role in the surveillance component but more work is needed in improving treatment uptake, both in terms of the threshold for treatment referral and perhaps also in regards to a more effective alternative to benzathine penicillin. We are currently investigating the role of cotrimoxazole in this regard. Although we found no discernible impact against scabies, we know that reducing scabies prevalence will further reduce pyoderma prevalence. The potential for oral ivermectin as a more acceptable alternative to community-wide use of scabicide cream, particularly for adult household contacts, needs to be investigated amongst Indigenous populations where scabies continues to be endemic.

Like most people living in remote Indigenous communities, as well as those working in primary health care, we acknowledge that much still needs to be done. However, on balance, we believe the results presented here are a good news story for local action to address a serious public health problem.

## Supporting Information

Checklist S1 CONSORT checklist.
(0.06 MB DOC)

Protocol S1 EAHSP study protocol 41, 17 May 2004, excluding some components for cultural, intellectual property, or commercial-in-confidence reasons.
(0.30 MB PDF)
