# Supplementary material for: A Regional Initiative to Reduce Skin Infections amongst Aboriginal Children Living in Remote Communities of the Northern Territory, Australia
Source: PLoS Negl Trop Dis. 2009 Nov 24;3(11):e554. doi: 10.1371/journal.pntd.0000554 (PMC2775159; doi:10.1371/journal.pntd.0000554)
Supplement: Protocol S1 — EAHSP study protocol 41, 17 May 2004, excluding some components for cultural, intellectual property, or commercial-in-confidence reasons. (0.30 MB PDF) [file pntd.0000554.s002.pdf]

East Arnhem Regional Healthy Skin Program

July 2003 – June 2007

A regional collaboration to reduce scabies, skin sores and associated chronic diseases, including rheumatic fever and renal disease, among Australian Aboriginal communities

Protocol ID 41
Version date 17 May 2004

Principal Investigators: Jonathan Carapetis[1] and Christine Connors[2,3]
Associate Investigators: Ross Andrews,[1] Norma Benger,[4] Bart Currie,[2,4] Lisa McHugh,[4] Melita McKinnon,[4] Colin Parker[5] and Shelley Walton[4]

1. Centre for International Child Health, Melbourne University Dept of Paediatrics and Murdoch Childrens Research Institute
2. Cooperative Research Centre for Aboriginal Health
3. Department of Health and Community Services, NT
4. Menzies School of Health Research, NT
5. Australasian College of Dermatologists

Executive Summary

Skin sores and scabies are directly linked to chronic diseases like rheumatic fever and streptococcal kidney disease, and may be linked to many other health problems including serious bacterial infections, childhood malnutrition, infections such as pneumonia and gastroenteritis, and antibiotic resistance. A program to control skin infections may therefore lead to dramatic health improvements. We have previously established successful models of community-based control of skin infections in Aboriginal communities. The challenges now are to ensure that these programs are sustainable, properly evaluated, of improved efficacy (using additional interventions and a regional approach), and integrated into routine service delivery.

The former Cooperative Research Centre for Aboriginal and Tropical Health (CRCATH) supported a qualitative evaluation of healthy skin programs. This research confirmed Aboriginal communities were enthusiastic supporters of a regional healthy skin strategy and identified numerous factors necessary for sustainability of these interventions. In response to this identified need, public health staff and researchers from the Menzies School of Health Research and the Murdoch Childrens Research Institute/University of Melbourne developed this proposal in collaboration with remote Top End communities and other stakeholders.

We propose to implement Healthy Skin Programs in approximately six Aboriginal Communities in the East Arnhem region. We will evaluate the program, it's impact and costs, and we will develop a model for similar programs that are sustainable and readily incorporated into routine service delivery throughout the Northern Territory.

A key component of the Program involves a single mass treatment for scabies of all residents in the region followed by regular monitoring of skin infections with treatment of individuals as new cases arise. Mass community treatment approaches have been shown to drastically reduce levels of scabies. By incorporating a model of regional (rather than individual community) treatment with continual monitoring, we expect that it will not be necessary to implement further mass treatment beyond the initial one-off activity. Additional measures will be used in each community to augment scabies control, including a selection of strategies to reduce streptococcal skin colonisation, reduce the impact of non-scabies related skin trauma, and provide early treatment to established skin infection.

We will develop a strategic plan for integration of the Program into routine service delivery in consultation with the community controlled medical service ([redacted]), the Northern Territory Department of Health and Community Services (NT DHCS), Department of Employment, Education and Training (DEET) and a range of other stakeholders.

Indications that a sustainable Healthy Skin Program will develop from this initiative are strong. The need for a regional approach was identified by a number of communities. Furthermore, we have received strong indications of support from communities in the East Arnhem region: [redacted], [redacted], [redacted], [redacted] and [redacted], as well as the [redacted]. Our team of experienced Indigenous researchers will work closely with local Aboriginal workers recruited from within each community. We will provide relevant training and support for each worker as well as for the wider community so that community enthusiasm will be maintained over the long term.

In addition to community support, the NT DHCS is also a supporter of the regional Healthy Skin Program and is encouraging other communities that wish to initiate or extend their own programs. Although our project team will not be able to directly provide support to these other communities, we have and will continue to provide advice when requested. As further indication of their support, NT DHCS have committed to provide a substantial "in kind" contribution related to staff participating in a number of implementation and data gathering activities (see Table 4).

The Healthy Skin Program has a strong emphasis on transferring skills and knowledge to the Aboriginal community workers and other community members. This should ensure that the Program is able to continue within these communities well beyond funded period without the high level of infrastructure and support required initially. We also have an emphasis on training of the Aboriginal staff, including the potential to employ Aboriginal laboratory staff and an Aboriginal doctoral student.

Our project team includes a central staff based at the Menzies School of Health Research in Darwin who will be the primary contacts for liaison with communities and for training and support of Aboriginal workers recruited from within each participating community. The Project Coordinator, [redacted], is a registered nurse with several years experience in clinical research. [redacted] is a recent appointment and will compliment the skills of our two experienced Indigenous researchers, [redacted], Senior Indigenous Research Officer, and [redacted], Community Liaison Officer. The implementation and integration components of the project will be run out of the Darwin base.

Team members from the Murdoch Childrens Research Institute/University of Melbourne will provide support for the evaluation component of the project with Ross Andrews appointed as the Study Epidemiologist. The Australasian College of Dermatologists will provide a dedicated consultant and registrar to provide assistance in screening and treatment, help standardise diagnoses and train Indigenous staff in making diagnoses of scabies, pyoderma (skin sores) and fungal diseases.

[redacted]

[redacted]

Table of Contents

Executive Summary 2
Compliance with CRCAH research criteria 7
Background 9
Response to community need 9
Healthy Skin Programs 9
The Proposal 9
Rationale 10
Scabies and streptococcal skin sores in the NT 10
Relationship to chronic kidney and heart disease 10
Community-based control of scabies and other skin infections 11
Objectives 15
Detailed Project Outline 15
Implementation 16
Consultation, planning and base line data collection 16
Recruitment and training 16
Treatment and monitoring 17
Additional “optional” measures 18
Management of scabies and skin sores 19
Annual health festival 20
Reporting 20
Evaluation 21
Community response 21
Ongoing monitoring of scabies and skin sores 21
Enhanced surveillance and record review 22
Laboratory surveillance for streptococcal sore throat and antibiotic resistance 22
Integration 23
Expected Outcomes 25
Indigenous Training and Research Capacity Building 26
Training of Medical Students 27
Governance 29
The Partners 29
Steering Committee 29
Project Team 30
Task Force Groups 30
Planning Groups 30
Program Budget 32
Appendix 1: Consultations and briefings undertaken for CRCAH funding submission 35
Appendix 2: Letters of support and confirmation of funding for the Healthy Skin Program 36
Appendix 3: Response to external reviewers’ comments from the Ian Potter Foundation 37
Appendix 4: Consent Forms, Data Collection Forms & Plain Language Statement 39
Appendix 5: Evaluation Framework 40
Appendix 5 Glossary and Abbreviations 45
References 46

List of Tables

Table 1: Scabies prevalence in selected community healthy skin programs 13
Table 2: Intervals between screening and scabies prevalence 13
Table 3: Healthy Skin Program Budget: expenditure and direct cash contributions 33
Table 4: Healthy Skin Program Budget: “In-kind” contributions 34

Compliance with CRCAH research criteria

1. Potential outcomes

This proposal will determine the costs and benefits of a health intervention (Healthy Skin Programs) and how the intervention can be applied in practice. All of the stakeholders who would eventually take responsibility for application of the intervention in routine service are represented in our study team. The research itself will have direct health gains (by improving the delivery of Healthy Skin Programs in the study communities) and will directly lead to wider application of the intervention in other Aboriginal communities. A specific task force will be charged with determining how the intervention will be integrated into routine service delivery, and we have commitments from NT DHCS and others to explore strategies for incorporating the program into policy and service guidelines. The development of Indigenous research and health service-delivery capacity is a key element of the proposal.

The expected outcomes include substantial reductions in scabies and skin sore prevalence, broader health effects relating to chronic diseases, acute illness presentations and antibiotic use, improvements in Indigenous research and health service-delivery capacity, and establishment of models for incorporating Healthy Skin Programs into existing health service delivery.

2. Robustness

Our research team has an excellent record of research in Aboriginal health and is backed by organisations experienced in providing administrative and infrastructure support to Aboriginal health research projects. The Program includes a high proportion of Indigenous staff, both as central support/research staff and within the communities. In addition to this, local planning groups are an integral component of the Program ensuring local people are involved in the decision-making processes.

This proposal has a high probability of success for a number of reasons. It is a natural extension of existing healthy skin programs, which have previously been shown to be successful in a number of communities. The novel elements of this research (including strategies in addition to scabies control and a regional approach) will ensure that the health gains are greater than previously demonstrated, and that they are quantified. Moreover, the previous CRCATH-supported Healthy Skin Feasibility Study confirmed the support of Aboriginal communities for a regional approach to coordinated Healthy Skin Programs, and identified several key elements necessary to ensure their long-term sustainability, all of which are incorporated into this proposal. Finally, our plan to transfer outcomes into routine service delivery is rigorous and has the support of relevant stakeholders.

We have a clear set of milestones detailed in the proposal, and the Steering Committee will regularly review our progress. The proposal has been peer reviewed by the Ian Potter Foundation and we have attached our responses to those comments (see Appendix 3).

3. Strength of research connectivity

This proposal involves an extensive network of Aboriginal communities, partner agencies and funding bodies (including industry), all of which are represented on the Steering Committee. We have held discussions with many individuals and organisations at all of these levels (see Appendix 1) and obtained numerous letters of support (see Appendix 2). Integration into routine service delivery is one of three key aspects of this proposal. We also include a section dealing with dissemination of the findings of this research.

The CRCAH partners directly involved in the project include MSHR, University of Melbourne, NT DHCS, OATSIH, DEET NT. Further involvement of the following partners is also expected: Charles Darwin University, Department of Community Development, Sport and Cultural Affairs.

The project will primarily address CRCAH Theme 4, with particular relevance to Research Topic 4.2.2 (diagnosis, prevention, treatment and control of scabies) and Research Area 4.1 (chronic diseases). The microbiological component of the Healthy Skin Program will be relevant to Research Topic 4.2.1 (vaccines for GAS and scabies). In addition, our research crosses Theme 1 through Research Topic 1.3.2 (workforce, training, recruitment, retention and performance) and Theme 2 through Research Topics 2.2.2 (strengthening families and communities), 2.3.1 (sustainable interventions for chronic diseases at the population level), 2.4.1 (risks in the physical environment), and 2.4.2 (living skills and behaviours in relation to health outcomes).

4. Value/cost

[redacted]

5. Relationship with CRC Business Plan

The proposed research is consistent with all aims and objectives of the CRCAH. Specifically, this is strategic research on health determinants and health conditions and will have input into health system development. We have a rigorous plan for transferring research outcomes into policy and practice, we will build on existing Indigenous research and health service-delivery capacity. The research will be high quality with extensive research partnerships and substantial Aboriginal participation and control.

Background

Response to community need

A feasibility study was conducted during 2000/2001 into the options for a coordinated Healthy Skin program across the Top End.[1] The main outcome was that communities that had successfully implemented their own programs were keen to participate in coordinated programs with related communities. There was widespread interest amongst other communities who had not run the program, but they wanted to develop their own experience prior to committing to a broader coordinated program. Many communities had been unable to effectively evaluate their program, but identified sustainability as an issue for further research that should be a prerequisite to extending the program.

Healthy Skin Programs

A number of community-based healthy skin programs have been implemented in the Northern Territory and have been successful in controlling scabies and related skin infections. They have shown that such programs are feasible in the NT and are supported by Aboriginal communities. However, previous programs have been limited to single communities with varying levels of success. There is an urgent need for a coordinated regional approach to healthy skin programs in Aboriginal communities including:

- A detailed evaluation of the health impact;
- Identification of factors that promote long-term sustainability; and
- How to translate such interventions into routine service delivery.

We believe that a regional program should be able to demonstrate, not only a reduction in the burden of scabies and streptococcal skin sores, but also broad health benefits, including primary prevention of some chronic diseases that occur at particularly high rates in Aboriginal communities.

The Proposal

Our proposal is based on the premise that there is a valid rationale for promoting Healthy Skin Programs in that they directly reduce rates of scabies and skin sores. However, demonstrating the likely broader health benefits would provide added impetus for their introduction as part of routine service delivery. Moreover the factors that promote long-term sustainability of these programs have yet to be fully explored.

We propose a coordinated approach to introducing Healthy Skin Programs in a number of Top End Aboriginal communities, to be accompanied by a detailed evaluation of their impact on diverse indicators of health outcomes, and of the factors that promote sustainability. In addition, we will focus on mechanisms for disseminating the results, and for integrating these programs into routine service delivery.

Rationale

Scabies and streptococcal skin sores in the NT

The most important skin infestations and infections among Aboriginal communities in central and northern Australia are scabies and streptococcal skin infection.[2] Scabies is currently endemic in many remote Northern Territory Aboriginal communities, with prevalence in children up to 50% and in adults up to 25%.[3,4] However, scabies has only become endemic in many remote Aboriginal communities over the last several decades.[5,6] Household overcrowding, access to adequate quantities of water, high humidity, education and implementation of personal hygiene are all important factors which increase the risk of these infections.[7,8]

Molecular typing of scabies mites has shown multiple overlapping epidemic cycles.[9] Apart from the individual discomfort caused by scabies, it underlies 50% to 70% of streptococcal skin infection.[4] Streptococcal skin infections are also endemic in these communities, with prevalence in children often exceeding 70%.[4]

Relationship to chronic kidney and heart disease

There is a link between scabies and skin infections in childhood, and the extreme rates of end-stage renal failure in Aboriginal adults. Acute post streptococcal glomerulonephritis (APSGN) is an inflammatory disease of the kidneys that periodically causes outbreaks involving hundreds of children in multiple Top End communities.[10,11] The risk of developing APSGN during an epidemic is increased by five for children with skin sores and doubled for children with scabies.[12] A recent study followed up a cohort of Aboriginal children in the NT an average of 15 years after two APSGN epidemics had struck their communities. The study found APSGN in childhood increased the risk of adult renal disease by six (6) times.[13]

Streptococcal skin infection is also linked with acute rheumatic fever (ARF) and rheumatic heart disease (RHD). ARF and RHD occur at the highest rates in the world in the Aboriginal population, and are important causes of premature mortality.[14,15] Whereas the cause of ARF has traditionally been attributed to group A streptococcal (GAS) infection of the upper respiratory tract, recent work by us and others has raised the possibility that skin infection with GAS may be involved in the pathogenesis of ARF.[16-18] The possibility that not all ARF is due to GAS infection of the throat is supported by a recent large randomised controlled trial in New Zealand where a sore throat-based primary prevention of ARF did not significantly reduce ARF incidence (Prof D Lennon, unpublished data presented at ICAAC and WSPID, 2002).

The evidence suggests that controlling skin infections will not only lead to reduced morbidity from scabies and skin sores, but is also likely to lead to reduced rates of APSGN and consequently chronic renal failure, as well as reduced rates of ARF and RHD. In addition, there is also reason to believe that the public health impact of a coordinated skin control program will be even more substantial. Skin infections are the major underlying cause of serious bacterial infections (GAS and *Staphylococcus*). These infections are very common in Aboriginal communities and have high mortality.[19,20] Moreover, children in Aboriginal communities commonly have many skin sores (rather than the one or two sores that would raise alarm in other settings). It is not difficult to envisage that the bacteria from these skin sores may cause a proportion of the cases of pneumonia and febrile illness that are common reasons for Aboriginal children attending clinics or being admitted to hospital.

We postulate that chronic, persistent and severe skin infection may have effects on children's immune systems and general state of well-being, which lead to the inability to fight other infections (e.g. leading to increased frequency and severity of some of the more common childhood infections such as pneumonia or gastroenteritis) and also impairs children's appetites, exacerbating malnutrition.

Skin infections are among the most common reasons for childhood presentations to clinics in Aboriginal communities, and consequently for the prescription of antibiotics.[21] In addition to the financial cost of these treatments, they undoubtedly contribute to the spreading epidemic of antibiotic resistance, particularly among pneumococcal bacteria, in Aboriginal communities.

As outlined below, we have conducted healthy skin programs in a number of individual communities. The involvement of the whole community has been an important component of these programs resulting in a range of community initiated interventions such as community clean-up days where community staff have provided clotheslines, laundry powder, assistance for older people and rubbish collection. Schools, community stores, outstation councils and women's centres have all been involved within different communities. The effect on the health of community members of this sort of community mobilisation could potentially be substantial. At present, data to support these assertions are only anecdotal. During a two-year scabies control program in one Aboriginal community,[4] we noticed an apparent dramatic reduction in the proportion of children with malnutrition and the number with serious medical complaints.

We believe that a program to reduce the prevalence and severity of skin infections may have very broad impacts on child and adult health, including:

- *Direct reduction of morbidity from scabies and skin sores,*
- *Reduced incidence of acute post streptococcal glomerulonephritis and acute rheumatic fever, leading to reduced morbidity and mortality from renal disease and rheumatic heart disease,*
- *Reduced incidence and mortality from severe streptococcal and staphylococcal infections,*
- *Reductions of clinic presentations and hospitalisations for a wide range of illnesses,*
- *Improvements in childhood nutritional parameters,*
- *Reductions in overall antibiotic prescriptions, and*
- *Reductions in antibiotic resistance in circulating bacteria.*

Community-based control of scabies and other skin infections

Control of scabies appears to be critical in controlling streptococcal skin infection and its associated complications. Previous approaches aimed at controlling dog scabies have no role in controlling human scabies, as molecular typing methods have demonstrated that the scabies mite infecting people is a separate variant from dog scabies.[9] The accepted treatment for scabies involves applying an anti-scabicide cream or lotion for the individual patient and their close contacts.[22] In small indigenous communities with high population prevalence and extended family communal living, this traditional approach has not been successful in preventing the transmission cycle between people.

A successful model to control high rates of scabies in a community setting has been adapted from Panama and used effectively in several remote NT communities.[23] The Panama program involved simultaneous supervised treatment, with 5% permethrin cream (an effective anti-scabicide treatment), of all community members living on an island. Prevalence rates of scabies reduced from 33% of the population to very low levels of 1.5% that were sustained for three years. The prevalence of pyoderma (skin sores infection) in children decreased from 32% to 2% without use of specific antibiotics. Community workers provided regular screening of the population following the single treatment intervention, and supervised treatment for any new cases of scabies. The amount of permethrin used in the single community treatment was less than that used in a neighbouring island to treat individual cases and their contacts over a 12 month period. Unfortunately, political events in Panama interrupted ongoing monitoring of the program.

The first attempt in the Top End at reducing the community prevalence of scabies and skin sores was implemented at [redacted] in 1992 in response to a large epidemic of APSGN. The program focused on treating households with recurrent scabies and showed some success with a lower prevalence maintained for up to two years (personal communication S. Guthridge).

In 1994, we formally trialed an adapted Panama model at [redacted] and were successful in maintaining low rates of scabies and skin infection for up to five years.[4] The scabies rate decreased from 29% to below 10% at two years. Additionally, without specific antibiotic interventions, the prevalence of pyoderma in children dropped from 69% to around half that rate and the residual pyoderma was documented as significantly less severe using a “sore score” quantitative assessment. The main differences between the Panama and [redacted] interventions was that community members at [redacted] did not want supervised treatment and did not consider automatic treatment for all visitors appropriate.

Following the success of [redacted] program, guidelines for health staff were developed with NT Centre for Disease Control.[24] The key features of the program include:

- Community planning and collaborative effort;
- Development of local educational resources;
- Single whole community treatment day for scabies with permethrin (5%);
- Community “clean up” involving house cleaning, linen and rubbish collection;
- Emphasis on housing functionality and “washing kids” to maintain low rates of skin sores; and
- Regular monitoring of scabies and skin sores, usually in a target group such as children

These programs have since been implemented in a number of Top End communities where some small focus group interviews found a positive response to the programs. Different models have been adapted to local conditions, including senior women within the communities working with researchers to provide screening and community education. The main difference between communities has been the level of success in maintaining low scabies rates over time. Three communities that have successfully maintained low rates are [redacted], [redacted] and [redacted] (see Table 1). The common elements amongst these programs were ongoing surveillance, community awareness of rates of scabies and skin infections and regular input from workers based in Darwin with MSHR, CRCAH and/or NT DHCS. In contrast, for the other communities that have available data, the prevalence rates approached pre-program levels within seven months (eg. [redacted] and [redacted]).

Table 1: Scabies prevalence in selected community healthy skin programs

Community	[redacted]	[redacted]	[redacted]	[redacted]	[redacted]	[redacted]	[redacted]	[redacted]
Year	1992	1996	1998	1998	2000	2000	2000	2001
Population	450	250	1740[a]	723	2455	2085	1200	1100
Screened	----	62	326	189	217	513	94	103
Scabies prevalence	%	%	%	%	%	%	%	%
Initial screen	45	33	33	57	35	61	42	33
At 6 weeks	10	8	5	---	2.9	2.5	30	7.2
At 4 months	24	5	---	---	5	---	---	---
At 7 months	---	---	29	29	4.1	---	---	13.1
At 9 months	---	9	---	---	2.3	---	---	---
At 12 months	---	---	35.5	---	2.8	---	---	3.8
At 14 months	---	8	14	---	8.5	---	---	3.0
At 21 months	---	6		48	12.2	---	---	
At 24 months		7			9.9[b]			
At 28 months					5.0			

[a] population treated, not total population
[b] second community treatment day

Ongoing surveillance following the initial mass treatment appears to be an important factor in sustaining low rates of infection but the rationale behind this has not been explored. Results from [redacted] have shown that the majority of new cases of scabies are occurring as isolated cases, with a smaller proportion as recurrent cases. Mild scabies may be less infectious than anticipated, and regular screening intervals may detect new cases within a time frame that reduces the potential for transmission. The intervals for surveillance have varied between the three communities that have maintained low rates ([redacted], [redacted] and [redacted]). On average, screening for scabies and other skin infections has been conducted every 3.1 to 4.2 months in each community, with the interval for follow-up ranging from 1 to 7 months. In these three communities, the mean prevalence varied from 5.9 to 7.2% and there was a non-significant trend towards increasing scabies prevalence with increasing length of surveillance interval (Table 2).

Table 2: Intervals between screening and scabies prevalence

[redacted]		[redacted]		[redacted]	
Month (interval)	Scabies	Month (interval)	Scabies	Month (interval)	Scabies
0 (0)	33%	0 (0)	35.0%	0 (0)	33.0%
2 (2)	8%	1 (1)	2.9%	2 (2)	7.2%
4 (2)	5%	4 (3)	4.1%	7 (5)	13.1%
9 (5)	9%	6 (2)	2.3%	11 (4)	3.8%
14 (5)	8%	9 (3)	2.8%	14 (3)	3.0%
21 (7)	6%	12 (3)	8.5%		
		17 (5)	12.2%		
		21 (4)	9.9%		
		25 (4)	5.0%		
Average interval and prevalence					
(4.2)	7.2%	(3.1)	5.9%	(3.5)	6.75%

Objectives

The primary objectives of the regional Healthy Skin Program are:

1. To implement a coordinated regional approach to healthy skin programs in approximately six Aboriginal communities in the East Arnhem region.
2. To complete a detailed evaluation of the program's impact on diverse indicators of health outcomes, and of the factors that promote sustainability.
3. To develop a plan to incorporate these programs into routine service delivery.

Detailed Project Outline

The East Arnhem Region is an area where rheumatic heart disease is a major problem. A number of communities in the region, [redacted], [redacted], [redacted], [redacted], [redacted] and [redacted], have expressed an interest in participating in the regional Healthy Skin Program. An annual healthy skin day has been conducted in [redacted] for a number of years, whilst other communities in the region have conducted programs sporadically in the past.

Consultations have been held with individuals from communities and relevant policy makers and service providers from local health clinics in the region; the community controlled health service ([redacted]); the Department of Health and Community Services; the Department of Employment, Education and Training; and other relevant agencies (see Appendix 1). There is significant support for the program and including commitments negotiated with key stakeholders.

In collaboration with communities in the East Arnhem Region, we propose to conduct a coordinated Healthy Skin Program. By concentrating on one region, the project has an increased likelihood of success because reinfection due to mobility between communities will be minimised, and because the project will be recognised as a collaboration between multiple communities.

The program is structured into three distinct components:

- Implementation of the program within the participating communities;
- Evaluation of the program against specific outcomes; and
- Integration of the program into routine service delivery.

Implementation

Consultation, planning and base line data collection

Preliminary work on this project commenced in July 2003 following funding received from the Rio Tinto Aboriginal Foundation. This work has focussed on review of data from other healthy skin programs, development of protocols, databases, and preliminary discussions with key stakeholders, development of funding submissions and applications for ethics committee approvals.

The current phase, March 2004 – September 2004, will focus on recruitment of participating communities, establishment of local planning groups and baseline data collection. The consultation will emphasise that the Healthy Skin Programs will be owned and run by the community, with central staff playing only a support role. The central staff primarily responsible for support and liaison with community groups will be three experienced researchers: [redacted] (Project Coordinator), [redacted] (Senior Indigenous Research Officer) and [redacted] (Community Liaison Officer).

We will facilitate a joint meeting of all participating East Arnhem communities. At this meeting, we will outline the basic model for the Healthy Skin Program and the opportunities for communities to incorporate a range of other community driven initiatives into their own community program. The basic model for the regional Healthy Skin Program includes:

a. Participation and mobilisation of multiple community organisations to provide and support community education and health promotion about skin health and "washing kids" to reduce skin sores;
b. A one-off, mass treatment of scabies for the whole community using topical permethrin 5% cream;
c. Regular monitoring of skin health in each community, concentrating primarily on children but also including families identified initially as having high scabies prevalence (Appendix 4); and
d. Regular reporting/feedback to communities and other key stakeholders.

The community-specific plan will be developed in collaboration with the local planning group in each community. The plan will outline the additional measures that the community wants to undertake. Communities will have the opportunity to review the methods and results from other healthy skin programs such as those conducted at [redacted], [redacted] and [redacted] and to benefit from the experience of these communities. For example [redacted] Regional Council have commissioned a pictorial presentation which highlights the collaboration between various organisations as part of their "Puy Puy Scabies" program in [redacted]. Health promotional materials will be developed in preparation for the program.

Recruitment and training

We aim to recruit and train at least two local Aboriginal community workers in each participating community (total 0.5 FTE per community). This is an enhancement on previous Healthy Skin programs. These workers will not be drawn from existing service-delivery staff (e.g. AHWs in community clinics) except if it is agreed by all concerned that this is appropriate, and not to the detriment of routine service delivery. A potential model that is used by other projects based at MSHR is to employ retired health workers or other community workers with some health care experience.

The primary role of the local Aboriginal Community Workers will be to support ongoing monitoring and management of scabies and skin sores, maintain an interest and awareness of Healthy Skin and to work with community organisations to improve health hardware. Shortly after recruitment, all workers will be invited to a two-day workshop in Darwin that will combine training on the health aspects of the diseases addressed in the Healthy Skin Program with a broad based discussion with the project team of issues related to the program. Aboriginal Community Workers will also receive ongoing in-service workshops, particularly related to the health promotional and environmental health aspects. Further detail related to the recruitment and training of Aboriginal community workers is included in the section titled *"Indigenous training and research capacity building"*.

The health promotional aspects of the work undertaken by the local Aboriginal Community Workers will reinforce understanding by community members of the link between skin infections and other diseases, particularly rheumatic fever. Where possible in some communities – either because the community worker has sufficient health training or because of the interest of other health staff – regular contact will be made with rheumatic fever patients in the community, discussing the disease and the importance of adherence to prevention programs (such as antibiotic secondary prophylaxis). The MSHR Rheumatic Heart Disease Education Package (booklets and videos) developed by us will be an invaluable tool to assist in this health promotional component. In addition, the Top End Community Paediatrician (Dr Keith Edwards) is presently heading a national task force that is developing a "tool kit" that can be used by communities to improve adherence to secondary prophylaxis. During the course of our study, we may be able to implement this tool kit and potentially conduct a formal evaluation of it.

The budget allocation includes an essential allowance for training and peer support for project staff and also includes employment of a Aboriginal laboratory scientist, and a Aboriginal postgraduate student. Annual meetings of the project team will be held to enable review of progress, peer support and discussion of relevant issues.

Treatment and monitoring

The combination of a single mass treatment followed by ongoing monitoring has been successful and sustainable in communities that have had community leaders as the key providers of Healthy Skin information. The additional support for ongoing community surveys will help to ensure a continued focus on skin health.

A Healthy Skin Day will be conducted in each participating community to provide a one-off mass treatment of scabies for the whole community using topical permethrin 5% cream. As noted above, the Healthy Skin Day will be incorporated into a community-specific plan. We anticipate that communities may wish to conduct a broader health festival, in a similar fashion to that conducted at [redacted]. We will provide intensive support in the lead up to and on the day of the mass treatment for scabies. We expect the planning, support and implementation of this phase will be conducted over a five-month period from May 2004 – September 2004 during which we expect central staff will spend an average two weeks per month in the participating communities.

Prior to the Healthy Skin Day, baseline rates of scabies and skin sores will be ascertained through a combination of methods including assessment of period prevalence from existing data sources (clinic audits, Growth Assessment and Action (GAA) program, school screening) and an assessment of point prevalence during mass screening conducted prior to the Healthy Skin Day (see Appendix 4). We will also analyse baseline data from other communities that have conducted healthy skin days to assess the impact of seasonality on prevalence.

After the Healthy Skin Day, we will continue to monitor scabies and skin sores through existing data sources (clinic audits, GAA program data, school screening), rescreening in the lead up to the annual health festival in some communities, and active follow-up of preschool aged children by members of the project team. We will standardise our methods for assessment of the severity of scabies and skin sores to ensure inter-operator consistency among project team members.

The active follow-up of preschool aged children will be conducted by the local Aboriginal Community Workers every three to four months. Support staff based in Darwin will visit each community to assist with re-screening during the first year but, thereafter, visits will reduce in frequency and duration, focussing on peer support for the Community Workers and collection of data for evaluation of the program. The data collected during the regular re-screening will be compared with data routinely collected through the GAA program for each of the communities (see evaluation)

We hope to establish if the GAA data alone can be used for effective monitoring of scabies and skin sores. Rather than conduct regular re-screening, we think one option would be for the local Aboriginal Community Workers to focus on assisting the clinic in the conduct of the GAA program, particularly with respect to scabies and skin sores. The workers could assist by following-up children who had not yet presented at the clinic for their scheduled visit. Two communities outside the East Arnhem Region, [redacted] and [redacted], have a sustained history of involvement in healthy skin programs. We believe these communities would be ideal sites to trial this method. If successful, it could then be implemented within the participating East Arnhem communities.

Whilst the GAA program is an existing system that may be well suited to ongoing monitoring of scabies and skin sores in children under five years, school based screening may provide a similar opportunity for monitoring scabies and skin sores in older children. This option has been discussed with both NT DHCS and DEET and there is enthusiasm for the idea. School based screening has not been rigorously applied and the Healthy Skin Program may present an opportunity for reinvigorating this process in communities.

Additional "optional" measures

In addition to the core elements of mass scabies treatment, ongoing re-screening and targeted treatment, we will discuss with each community a number of other measures that may potentially be added to the local Healthy Skin intervention. These measures are designed to augment the efficacy of scabies control on skin sore prevalence by reducing streptococcal skin colonisation, reducing the impact of non-scabies related skin trauma, or providing early treatment to established skin infection.

Hygiene related issues are an essential element of Healthy Skin Programs, which have not been fully integrated into previous programs. In our discussions, families have had a good understanding of the causes of skin infections. The Healthy Skin Program will work closely with local Councils, environmental health officers and others to develop infrastructure that supports healthy behaviour. Suggestions for particular interventions may also come from within the community, but potential hygiene related interventions include:

- Campaigns to promote the daily washing of children with soap and water.
- A focus on community cleanliness and hygiene. This may range from community clean-up campaigns through to measures to improve the understanding of home cleanliness.
- Involvement of environmental health officers in the above strategies, to provide advice and also to ensure the availability and maintenance of hygiene infrastructure (such as water supplies to houses).
- Community education about disinfection and protection of cuts, abrasions and insect bites.

Other measures could include strategies to reduce the prevalence of insect bites (e.g. permethrin-impregnated bed nets). Antibiotic treatment of children with residual skin sores, using either intramuscular benzathine penicillin G or supervised oral treatment with an appropriate anti-streptococcal antibiotic could also be considered.

Management of scabies and skin sores

Severe cases of scabies and skin sores detected following the initial mass treatment will be referred to the health clinic for management. This will include use of antibiotics for the treatment of skin sores when appropriate (according to Top End standard treatment guidelines), re-treatment of those with persistent scabies and their families, and, if appropriate, the use of ivermectin for treatment of those with crusted scabies (this would only be done in consultation with specialist medical staff in Darwin).

Monitoring of scabies resistance is an integral part of the project. We have consulted with the investigators involved in the recently-funded NHMRC project grant collaboration between MSHR and QIMR looking at mechanisms of resistance to scabicides in scabies mites (Drs Walton, Currie and McCarthy). We have agreed to collaborate, and will be working closely together to monitor any emerging resistance. This will consist of regular collection of scabies mites in selected communities participating in the Healthy Skin project, with laboratory testing for resistance. Mites will be collected from a sample of people in communities at baseline (to determine baseline resistance rates), and then from people with recurrent or persistent scabies subsequently (to determine if persistent or recurrent scabies is related to resistance). If nearby communities not participating in the Healthy Skin project are planning scabies control programs, we will also approach them for permission to collect scabies mites (with ethics committee approval and appropriate informed consent) in order to determine if any resistance found in Healthy Skin communities is related to the Healthy Skin program per se, or instead a phenomenon occurring more widely in the region.

The collection of scabies mites will be supervised by the dermatologists and dermatology registrars participating in the program, together with members of the team who have had training in mite collection from Prof David Taplin in Panama ([redacted] and [redacted]). Mites are easily collected from bedclothes and skin flakes of people with severe or crusted scabies. Collection of mites from people with less severe scabies is more difficult, and relies on carefully picking the mite using a magnifying glass, drop of oil and a small needle. If the operator is well trained, this procedure is painless and relatively easy. We will ensure that all operators are properly trained for this purpose.

The Australasian College of Dermatologists will support the project by allocating a consultant and a registrar to each community. In addition to providing expert advice regarding the management and treatment of scabies and skin sores, they will also provide advice for other skin conditions such as tinea. By providing a service for advice and treatment for tinea we will broaden the focus of the Healthy Skin Program because this has been an issue of concern to adults in other communities.

Annual health festival

We will encourage all communities to have an annual health festival, incorporating a Healthy Skin Day. Such events would be an opportunity to highlight progress in the prevention of scabies, skin sores and various other outcomes. An annual check of scabies and skin sores would be offered to the whole community with central staff available to assist in a large scale re-screening such as this. We would however not recommend repeated mass treatment on an annual basis.

In our experience, population based screening for scabies and skin sores among older age groups has been problematic due to poor response rates. We hope to improve participation rates for the older age groups by providing a broader range of services at the annual health festivals including advice on other skin conditions such as tinea, and screening for heart murmurs. All age groups will be able to participate and only summary de-identified data will be recorded specifying the date of screening, age, sex, skin condition or presence/absence of heart murmur (Appendix 4).

Any people with heart murmurs detected during the project will be counselled initially by study staff and, with the individual's permission, their case will be notified to the local District Medical Officer (DMO). We will follow the standard procedure recommended in the Best Practice Guidelines of the NT RF/RHD Control Program, which is that the murmur should first be confirmed by a qualified medical practitioner and then the person referred for echocardiography. We will ensure that any cases of RHD are entered onto the RHD register and referred for appropriate follow-up. Cases of other heart diseases will also be referred, via the DMO, for specialist review.

Reporting

Throughout the project, regular progress reports will be provided to communities and other key stakeholders. This will include graphs showing the trends in the prevalence of scabies and skin sores immediately following the monitoring conducted by the Community Workers and the central staff, as well as an annual report.

Evaluation

Evaluating the outcomes of a regional Healthy Skin Program is one of the essential elements of this project. We designed the evaluation as a separate component so that it does not unnecessarily burden the implementation component, and to ensure its integrity. We will utilise data collected during the implementation stage as well as other supplementary data sources. The program will be comprehensively and continuously evaluated by assessing:

- Community satisfaction and involvement;
- Program implementation strategies;
- Rates of skin sores and scabies from regular surveys;
- The prevalence and rates of change of particular types of streptococci in communities;
- Adherence to rheumatic fever secondary prophylaxis;
- Rates of acute post-streptococcal glomerulonephritis and incidence of rheumatic fever;
- Clinic presentations and hospitalisations due to streptococcal and staphylococcal diseases, pneumonia, gastroenteritis, and all causes;
- Antibiotic prescriptions for skin infections and all causes;
- The effect on nutritional parameters (weight for age, weight for height).

We have developed an evaluation framework (Appendix 5), which summarises the proposed methodology for each of the specific program objectives.

Community response

A detailed evaluation of program assumptions and implementation will be undertaken in at least three of the participating communities. We will endeavour to canvass a wide range of opinion from family groups and organisations, including those who did not participate or rarely participated in the Program.

We recognise that there are a number of key stakeholders, particularly local Aboriginal people with relevant research and other experience, who could provide advice on the best and most appropriate methods for gathering this information and assist in this process. [redacted] We propose to utilise a range of information gathering methods including questionnaires, focus groups, and individual interviews, to assess levels of understanding about the diseases being targeted and about the program, community satisfaction, and to identify particular issues that may relate to long-term sustainability.

Ongoing monitoring of scabies and skin sores

Utilising baseline data collected from each participating community and the data collected from the ongoing monitoring, we will compare the prevalence and severity of scabies and skin sores over time for each participating community. As noted previously, we will compare the data we collect during our ongoing monitoring against that collected in the clinic records under the GAA program.

Our re-evaluation of surveillance data from previous Healthy Skin Programs identified a potential source of bias where a number of children assessed initially were not reviewed at subsequent surveillance visits. Therefore, we will identify a specific cohort of children in each community to be followed up at subsequent visits, and make every effort to review all of these children. High mobility of communities has meant that such approaches have been difficult to maintain in the past. However, by taking a regional approach, we will be able to follow-up children who move between these communities much more effectively. As a result, we will have two measures of scabies and skin sore rates: overall prevalence in children in the community, and prospective prevalence monitoring in a specific cohort of children.

We do not plan to monitor adults at each 3-monthly review, as previous experience has confirmed that this is successful in only a subset of adults. Instead, monitoring of adults will occur at the yearly health festivals. We hope that the inclusion of management and treatment of tinea and screening for heart murmurs will encourage more adults to participate.

Enhanced surveillance and record review

We will establish enhanced surveillance for rheumatic fever and post-streptococcal glomerulonephritis in each participating community. This will be done through provision of detailed education for local clinic staff and a protocol for evaluation of possible new cases.

We will also conduct a retrospective review of available data from medical records (augmented by a search of computer databases where these exist in individual communities) to determine the incidence of clinic presentations and hospitalisations among children in the previous two years due to specific causes (skin infections, scabies, respiratory illnesses, diarrhoea) or for any cause. Where possible, an estimate will also be made of the incidence of antibiotic prescriptions to children for all causes and separately for skin infections.

Laboratory surveillance for streptococcal sore throat and antibiotic resistance

In order to assess the impact of the Program against streptococcal and staphylococcal diseases, it is critical to closely monitor the bacteriological effects of the intervention. The aims of this component are to document any reductions in prevalence of streptococcal and staphylococcal skin infections, monitor for any changes in streptococcal throat carriage rates (because of the hypothesis that widespread skin infections may provide relative immunological protection from throat infection/carriage) and maintain surveillance for the emergence of new, potentially virulent strains. Our monitoring of the microbiological effects of the Healthy Skin Programs will be divided into two components:

1. In four communities, we will identify a cohort of 60 to 100 children in each community (selected according to community size: 60 children in communities with <800 residents, 100 children in communities with >800 residents; 50% pre-school age and 50% school age) who will have swabs taken of their throat and up to two skin sores every three months. In addition, 30 children in each community will have nasopharyngeal swabs taken every six months.
2. In two communities, we will conduct more intensive surveillance where we will aim to arrange for all children to have swabs taken of their throat and up to two skin sores every three months.

The swabs will be transported and cultured according to our existing protocols. Streptococcal colonies will be grouped and stored. Group A streptococcal isolates will be transported to our Melbourne laboratories for *emm* sequence typing. Group C and G streptococci will be assessed at regular intervals by pulse-field gel electrophoresis. Staphylococcal isolates will be compared using RFLP or other suitable PCR-based methods. A subset of streptococcal and staphylococcal isolates will also be tested for resistance to penicillin, erythromycin and (in the case of staphylococci) methicillin, to determine the effects of the program on emerging antibiotic resistance. The nasopharyngeal swabs will be cultured for pneumococci, *Haemophilus influenzae* and *Moraxella catarrhalis*. These isolates will be tested for antimicrobial resistance, with the aim of determining any effects of reduced antibiotic prescribing on antibiotic resistance in non-skin organisms.

Our hypothesis that skin infections are linked with rheumatic fever is based on the assumption that group A streptococcal pharyngitis is uncommon in Aboriginal communities. However, although we have clearly demonstrated low throat carriage rates of group A streptococcus, we have never conclusively demonstrated low rates of symptomatic pharyngitis. Therefore, in two communities, we will establish active surveillance to determine the incidence of streptococcal sore throat. This will involve throat swabs and a simple clinical data form to be maintained at the clinic for all children presenting with sore throat. In addition, regular medical chart audits will be conducted to determine the number of cases missed through clinic-based surveillance. Finally, at the re-screening visits, all school-age children and their parents will be questioned to determine the number of cases of sore throat in the previous month. These last data will be compared with the clinic-based data to determine to what extent the clinic data are affected by health-seeking behaviour (i.e. children with sore throats may not present to the clinic).

Integration

At the completion of this project, our aim is to have the findings translated into routine practice, and the results widely disseminated for others to use. The close collaboration with NT DHCS, a key partner in the Program and member of the Steering Committee, means that if the findings are compelling translation into routine service delivery is highly probable. NT DHCS currently provides assistance to individual communities wishing to implement their own program, and strongly supports the regional approach.

[redacted]

As noted in the implementation component, we will be trialling methods for integration of the program into routine service delivery from the outset of the project. The fourth year of the project will focus on finalising a strategic plan for long-term amalgamation of the program into routine service delivery. The plan will be developed in consultation with the NT DHCS and other key stakeholders. We will quantify the cost implications and the benefits that are likely to ensue, together with the impact on health policy. The plan will include guidelines and standard frameworks for introduction of Healthy Skin Programs in other communities in Australia and internationally.

Subject to available finances, other communities in the region will be approached to participate in the program during the final year (July 2006 – June 2007). The purpose will be to demonstrate the feasibility of an expanded program. These approaches will include a presentation about the aims of the program and the progress to date. Evaluation in these other communities will focus on the prevalence of scabies and skin sores, and on issues relating to community acceptance and sustainability.

In terms of dissemination of results, aside from normal channels (e.g. publication in peer-reviewed journals and presentations at conferences), we intend to present our results formally and informally to policy makers and public health officials involved in Aboriginal Health. This may take the form of a formal meeting specifically addressing the issue. We will commit to ensuring that any positive or negative findings are communicated to those who need to know the results.

Expected Outcomes

We have identified expected outcomes for each of the primary objectives of the Program as listed below:

1. *Demonstrate a reduction in scabies and skin sores on a regional basis*
   - Reduced prevalence of scabies among children in the participating communities from 30% (expected prevalence pre-program) to <10% at least 2 years following the intervention
   - Reduced prevalence of skin sores among children in the participating communities from 50% (expected prevalence pre-program) to <25% at least 2 years following the intervention
   - Reduced severity of skin sores among children in the participating communities from 40% classified as moderate/severe (expected pre-program) to <15% classified as moderate/severe at least two years following the intervention

2. *Demonstrate the broader public health effects of Healthy Skin Programs, particularly those relating to chronic diseases such as rheumatic fever and renal diseases*
   - Establish a baseline of streptococcal kidney disease incidence (including outbreaks) and monitor reductions in cumulative incidence following the intervention.
   - Establish a baseline of acute rheumatic fever incidence and monitor reductions in cumulative incidence following the intervention.
   - Establish a baseline of adherence to rheumatic fever preventive medication within a subset of participating communities and improve adherence to at least 80%.
   - Establish a baseline prevalence of rheumatic heart disease, serious streptococcal and staphylococcal infections (particularly bloodstream and bone and joint infections) and monitor the effects of the program
   - Establish baseline indicators of overall child health, and on specific aspects of child health such as nutrition, incidence of infectious diseases, clinic presentations, hospitalisations and antibiotic prescriptions and evaluate changes following the intervention

3. *Build on the existing Indigenous capacity by assisting in the development of new knowledge and skills to improve the health and well being of Indigenous communities*
   - Train at least two Aboriginal community workers in each of the participating communities in health intervention, community development strategies and research processes specific to Healthy Skin Programs
   - Improve awareness of issues relating to scabies and skin sores and the link between these and other health conditions
   - Develop approaches to target residual skin sores

4. *Establish the feasibility of incorporating Healthy Skin Programs into existing health service delivery.*
   - Determine the costs and benefits of regional Healthy Skin Programs, and develop strategies for integration into routine service delivery
   - Assess community satisfaction with the Program

Indigenous Training and Research Capacity Building

Community education and awareness raising is a major component of the Program and constitutes a key strategy for building community capacity to have ongoing participation in the management of Healthy Skin Programs in the future. The project team has a strong commitment to a process that builds on and adds to the capacity of the community to participate in research and knowledge transfer activities. We recognise that the success of the Program relies heavily on the willingness and capacity of local communities to be involved in the design and implementation of the Healthy Skin Program, including the research activities. A number of components have been built into the design and relevant links are being established to ensure on-going and extensive participation of Indigenous people in the research process.

There is a growing research capacity within some Indigenous communities. The project will aim to draw on that experience, particularly in relation to recruitment and training of Indigenous researchers, consultation with communities on key success factors for the project, and appropriate strategies for data collection.

A program of peer support and mentorship will be developed for the Aboriginal Community Workers and for other Indigenous project team members. As noted previously, initial training of the community workers will focus on the health aspects of the diseases addressed in this project, which will then be supplemented by ongoing in-service workshops related to health promotional and environmental health aspects. Those persons interested in working towards a formal qualification will be encouraged to do so. At the conclusion of the project, the collaborators will undertake to assist each of the workers to use their newly-acquired skills in their existing work, or in seeking new employment. We will instigate discussions with appropriate institutions about relevant study pathways. We will also seek assistance and advice from the CRCAH Training Unit and from the Centre for the Study of Health and Society at Melbourne University.

Other initiatives include the possibility of establishing a research mentorship for an Indigenous researcher/community worker participating in the Healthy Skin Program is being explored through the Centre for Remote Health’s Primary Health Care Research, Evaluation and Development Program. In addition, a post-graduate scholarship will be offered so that an Aboriginal student may study important laboratory and/or public health aspects of streptococcal disease and program evaluation. The student will receive supervision from senior project investigators.

Training of Medical Students

Medical students at the University of Melbourne undertake a one-year Advanced Medical Science (AMS) component, at the completion of which they receive a Bachelor of Medical Science degree. We propose to offer AMS electives for four of these students for two years of the program. The students will each spend two periods of 8 – 12 weeks in the NT.

To ensure that the students understand the importance of ongoing contact with communities, and in order for them to see some of the outcomes of their work, each student will be attached to a single community. The student will be partnered with a local Aboriginal health worker, and participate in community surveys, health promotion, scabies and skin sore treatments, data collection, and program evaluation. The student will also spend time in Darwin understanding the broader aspects of the program, including the laboratory components, and the evaluation aspects. At the completion of their attachment, the student will complete an AMS treatise, which should also include personal reflections.

Governance

The Partners

Northern Territory	Cooperative Research Centre for Aboriginal Health (CRCAH)
	Menzies School of Health Research (MSHR)
	Department of Health and Community Services (NT DHCS)
	Miwatj Health Aboriginal Corporation
	Remote Aboriginal communities of East Arnhem
Melbourne	Murdoch Children’s Research Institute (MCRI)
	Centre for International Child Health (CICH), University of Melbourne Dept of Paediatrics
Sydney	Australasian College of Dermatologists
Canberra	Office of Aboriginal and Torres Strait Islander Health

Steering Committee

[redacted]

[redacted]

[redacted]

Project Team

[redacted]

[redacted]

[redacted]

Task Force Groups

[redacted]

[redacted]

Planning Groups

[redacted]

Milestones
Milestones have been listed based on a draft timetable of events, however these will be subject to negotiation with the participating communities and will be revised accordingly.

19 February 2004
- Obtain ethics approval for formal commencement of community consultation, recruitment and training of community workers, and analysis of de-identified datasets

24 March 2004
- Submit final ethics application including detailed study protocol, consent forms and data collection forms

24 May 2004
- First East Arnhem Regional Healthy Skin Workshop involving the Steering Committee, representatives from [redacted], [redacted], [redacted], [redacted], [redacted] and [redacted], and project team members.

30 June 2004
- Confirm involvement of participating communities and establish local planning groups to commence planning for coordinated Healthy Skin Day
- Recruit community workers
- Develop plans to integrate monitoring into routine service delivery at [redacted], [redacted]
- Complete preliminary analysis of de-identified data
- Validate skin sore score

30 September 2004
- Complete baseline data collection at community level
- Conduct ongoing training of community workers and finalise planning for the Healthy Skin Day
- Conduct coordinated Healthy Skin Day in participating communities

30 September 2005
- Regular on-site visits (every 3 months) by central staff in Darwin to support community workers in ongoing monitoring of scabies and skin sores.
- Collect swabs, clinic audits, school screening in two communities
- Review data and integration plan from [redacted] and &[redacted]
- Conduct annual health festival (not mass treatment) and provide annual report on progress to date for each community

30 September 2006
- Ongoing monitoring conducted predominantly by community workers and incorporated within routine service delivery at community level in accordance with integration plan. Central staff role reduced to one of predominantly peer support.
- Maintenance of swab collection, clinic audits, school screening in two communities
- Conduct annual health festival (not mass treatment) and provide annual report on progress to date for each community

31 March 2007
- Ongoing monitoring of scabies & skin sores now incorporated within routine service delivery, supported by community workers.
- Data collection completed

30 June 2007
- Complete data analysis
- Provide final report to all participating communities on the results of the Healthy Skin program

Program Budget

[redacted]

[redacted]

[redacted]

Table 3: Healthy Skin Program Budget: expenditure and direct cash contributions

[redacted]	[redacted]	[redacted]	[redacted]	[redacted]	[redacted]
[redacted]	[redacted]	[redacted]	[redacted]	[redacted]	[redacted]
	[redacted]	[redacted]	[redacted]		[redacted]
	[redacted]	[redacted]	[redacted]	[redacted]	[redacted]
	[redacted]	[redacted]	[redacted]	[redacted]	[redacted]
		[redacted]	[redacted]		[redacted]
	[redacted]	[redacted]	[redacted]	[redacted]	[redacted]
[redacted]	[redacted]	[redacted]	[redacted]	[redacted]	[redacted]
[redacted]		[redacted]	[redacted]	[redacted]	[redacted]
[redacted]	[redacted]	[redacted]	[redacted]	[redacted]	[redacted]
	[redacted]	[redacted]			[redacted]
	[redacted]	[redacted]	[redacted]	[redacted]	[redacted]
[redacted]	[redacted]	[redacted]	[redacted]	[redacted]	[redacted]
[redacted]	[redacted]	[redacted]	[redacted]	[redacted]	[redacted]
	[redacted]	[redacted]	[redacted]		[redacted]
	[redacted]	[redacted]	[redacted]	[redacted]	[redacted]
[redacted]	[redacted]	[redacted]	[redacted]	[redacted]	[redacted]
[redacted]	[redacted]	[redacted]	[redacted]	[redacted]	[redacted]
[redacted]	[redacted]	[redacted]	[redacted]	[redacted]	[redacted]

Table 4: Healthy Skin Program Budget: "In-kind" contributions

[redacted]	[redacted]	[redacted]	[redacted]	[redacted]	[redacted]
[redacted]					
[redacted]	[redacted]	[redacted]	[redacted]	[redacted]	[redacted]
[redacted]	[redacted]	[redacted]	[redacted]	[redacted]	[redacted]
[redacted]	[redacted]	[redacted]	[redacted]	[redacted]	[redacted]
[redacted]	[redacted]	[redacted]	[redacted]	[redacted]	[redacted]
[redacted]					
[redacted]	[redacted]	[redacted]	[redacted]	[redacted]	[redacted]
[redacted]	[redacted]	[redacted]	[redacted]	[redacted]	[redacted]
[redacted]	[redacted]	[redacted]	[redacted]	[redacted]	[redacted]
[redacted]					
[redacted]					
[redacted]	[redacted]	[redacted]	[redacted]	[redacted]	[redacted]
[redacted]					
[redacted]	[redacted]	[redacted]	[redacted]	[redacted]	[redacted]
[redacted]	[redacted]	[redacted]	[redacted]		[redacted]
[redacted]					
[redacted]					
[redacted]	[redacted]	[redacted]	[redacted]		[redacted]
[redacted]					
[redacted]	[redacted]	[redacted]	[redacted]	[redacted]	[redacted]

Appendix 1:
Consultations and briefings undertaken for CRCAH funding submission

[redacted]

[redacted]

[redacted]

[redacted]

[redacted]

[redacted]

[redacted]

[redacted]

[redacted]

[redacted]

Appendix 2:
Letters of support and confirmation of funding for the Healthy Skin Program

[redacted]	[redacted]
[redacted]	[redacted]
[redacted]	[redacted]
[redacted]	[redacted]
[redacted]	[redacted]
[redacted]	[redacted]
[redacted]	[redacted]
[redacted]	[redacted]
[redacted]	[redacted]
[redacted]	[redacted]
[redacted]	[redacted]

Appendix 3:
Response to external reviewers' comments from the Ian Potter Foundation

1. How will the right balance be struck between the science, the public health and health service components of the work?

   Response:
   Striking a balance between science, public health and service components is at the core of the project. We have gathered the appropriate expertise in each area on the project team. The project is a partnership between organisations involved in all of these areas, and this is reflected in the makeup of the Steering Committee.

2. How will local ownership and control, which is essential to the success of the project, be achieved while preserving the capacity to evaluate and to identify the success factors that will need to be replicated to ensure transferability and sustainability?

   Response:
   Local ownership will be achieved partly by running this as a CRCAH project, which in itself is majority Aboriginal controlled. Our team includes Aboriginal Community Workers employed specifically as local representatives of the project. In each community, a local planning group will be established to have input and oversight into study activities on site. The Steering Committee will include a representative of the study communities. We have deliberately separated the implementation and evaluation components, to allow for an objective assessment of the impact of the intervention. The implementation component will be the core responsibility of the team based in Darwin and the study communities. The evaluation will be coordinated by the Melbourne-based team members (A/Prof Carapetis and Dr Andrews).

3. How might the activities of skin disease be integrated into other health education and health promotion activities already in place?

   Response:
   The ways in which skin control activities can be integrated into other health education and promotion activities will be answered during the course of the project. This is part of determining how best to integrate Healthy Skin Programs into routine service delivery, which we have identified as a major outcome of the project.

4. How might intersectoral linkages (eg with schools) be made at community level?

   Response:
   The intersectoral linkages are also part of the project. This will be possible in that Healthy Skin Programs entail the mobilisation of the whole community. Everyone is involved, as opposed to many other public health projects, which only involve subsets of high-risk individuals. We have a track record of working with schools and other community groups in Healthy Skin Programs in [redacted], and the best ways that this can be done sustainably will be addressed during the project. In recognition of the importance of other service providers, we have already held productive consultations with the Education Department and Environmental Health.

5. What will be the model for managing the complex, inter-organisational collaboration required in this project (for example will there be a Memorandum of Understanding between the various parties to specify respective expectations, commitments and obligations)?

   Response:
   The management model is outlined in our proposal and consists of the Project Team (with three Task Force Groups to deal with implementation, evaluation, and integration) and a Steering Committee. We believe that this model, in which the major stakeholders are represented on the Steering Committee, will ensure that the inter-organisational collaboration is managed appropriately, and that formal contracts or Memoranda or Understanding between partners will not be necessary. However, we are happy to be further advised on this by the CRCAH.

6. Suggestion from [redacted] to consider auspicing a two day workshop in Darwin that would bring the partners, including funding bodies, to explore some of the aforementioned issues

   Response:
   The suggestion of a two day workshop in Darwin is a good one. We have budgeted for an initial face to face meeting of the Steering Committee and also a face to face meeting of the entire Project Team (including Aboriginal Community Workers).We propose to hold these two meetings simultaneously, with an initial combined workshop followed by separate meetings of the Steering Committee and Project Team.

Appendix 4:
Consent Forms, Data Collection Forms & Plain Language Statement

Appendix 5:
Evaluation Framework

The primary aims of the Healthy Skin Program are:
1. To implement a coordinated regional approach to healthy skin programs in approximately six Aboriginal communities in the East Arnhem region.
2. To complete a detailed evaluation of the program's impact on diverse indicators of health outcomes, and of the factors that promote sustainability.
3. To develop a plan to incorporate these programs into routine service delivery.

This document outlines the framework for the evaluation component, listing the key outcomes and a brief description of the proposed methodology for a series of specific program objectives. The evaluation will be an adjunct to the Healthy Skin Program and will be conducted within the following timetable:

Date	Activity
Jan 2004 – Mar 2004	• Submit preliminary ethics approval for confirmation of community participation, recruitment & training of community workers, analysis of de-identified data<br>• Project team planning meeting to review evaluation framework (objectives, endpoints, methods) and develop a workplan for all aspects of the Healthy Skin Program<br>• Commence consultation with East Arnhem communities re participation<br>• Develop plans to integrate monitoring into routine service delivery at [redacted] (community workers assist health clinic with GAA program)<br>• Commence preliminary analysis of de-identified baseline data<br>• Submit final ethics application including detailed study protocol, consent & data collection forms
Apr 2004 – Sep 2004 (6 months)	• Confirm East Arnhem community participation, establish local planning groups<br>• Recruit & train community workers<br>• Commence preliminary analysis of de-identified baseline data<br>• Validate skin sore score<br>• Subject to ethics approval commence baseline data collection at community level (clinic audits, screening)<br>• Anticipate coordinated mass treatment in participating communities (Sep 2004)
Oct 2004 – Sep 2005 (12 months)	• Regular on-site visits (every 3 months) by central staff in Darwin to support community workers in ongoing monitoring of scabies & skin sores<br>• Swabs, clinic audits, school screening in two communities<br>• Planning for annual health festival in September 2005 (not mass treatment)<br>• Review data & integration plan from [redacted]
Oct 2005 – Sep 2006 (12 months)	• Ongoing monitoring of scabies & skin sores predominantly conducted by community workers and, where possible, incorporated within routine service delivery at the community level as per integration plan<br>• Continue collection of swabs, clinic audits, school screening in two communities<br>• Central staff in Darwin shift from hands-on role to predominantly peer-support. Includes brief 6 monthly on-site visit and presentation of progress report coinciding with annual health festival in September 2006
Oct 2006 – Mar 2007 (6 months)	• Ongoing monitoring of scabies & skin sores now incorporated within routine service delivery, supported by community workers<br>• Data collection completed by March 2007
Apr 2006 – Jun 2007 (3 months)	• Completion of data analysis<br>• Final report presented to all participating communities

Objectives, Outcomes and Methodologies

1. Demonstrate a reduction in scabies and skin sores on a regional basis

Outcomes

- Reduced prevalence of scabies among children in the participating communities from 30% (expected prevalence pre-program) to <10% at least 2 years following the intervention
- Reduced prevalence of skin sores among children in the participating communities from 50% (expected prevalence pre-program) to <25% at least 2 years following the intervention
- Reduced severity of skin sores among children in the participating communities from 40% classified as moderate/severe (expected pre-program) to <15% classified as moderate/severe at least two years following the intervention

Methods

- Baseline rates will be ascertained in the lead up to the Healthy Skin Day through a combination of methods including assessment of period prevalence from existing data sources (clinic audits, Growth Assessment and Action (GAA) program, school screening) and an assessment of point prevalence during mass screening conducted prior to the Healthy Skin Day.
- Baseline data from other communities that have conducted healthy skin days will be analysed to assess the impact of seasonality on prevalence.
- Post intervention rates will be assessed using existing data sources (clinic audits, GAA program data, school screening), rescreening in the lead up to the annual health festival in some communities, and active follow-up of preschool aged children by members of the project team.
- Methods for assessment of the severity of scabies and skin sores will be standardised to ensure inter-operator consistency among project team members.

2. Demonstrate the broader public health effects of Healthy Skin Programs, particularly those relating to chronic diseases such as rheumatic fever and renal diseases

Outcomes

- Establish a baseline of streptococcal kidney disease incidence (including outbreaks) and monitor reductions in cumulative incidence following the intervention.
- Establish a baseline of acute rheumatic fever incidence and monitor reductions in cumulative incidence following the intervention.
- Establish a baseline of adherence to rheumatic fever preventive medication within a subset of participating communities and improve adherence to at least 80%.
- Establish a baseline prevalence of rheumatic heart disease, serious streptococcal and staphylococcal infections (particularly bloodstream and bone and joint infections) and monitor the effects of the program
- Establish baseline indicators of overall child health, and on specific aspects of child health such as nutrition, incidence of infectious diseases, clinic presentations, hospitalisations and antibiotic prescriptions and evaluate changes following the intervention

Methods

- Existing data sources collated by NT DHCS will be analysed to determine baseline rates and monitor cumulative incidence following the intervention among participating communities and among other Top End communities by age group over equivalent time periods (two years before versus two years after the intervention).
- Data sources include notifications of acute post streptococcal glomerulonephritis, the rheumatic fever register, the rheumatic heart disease register, the hospital admissions database, data from school based screening which incorporates urine and heart screening of 10 year olds, GAA data collected for children aged 0-2 years which will be utilised for assessment of nutritional status, and data on the volume and type of antibiotics supplied to individual communities.

- Clinic audits will be conducted to ascertain the overall number of clinic presentations for any reason, clinic presentations for infectious diseases, referrals for hospitalisation and to search for undiagnosed cases among persons aged up to 30 years during equivalent pre and post intervention periods.
- The possibility of including a heart murmur survey pre and post intervention for persons aged up to 30 years is being considered, perhaps as part of a women's and men's health week.
- Two communities will have an enhanced focus on rheumatic fever and on the impact of the program on group A streptococcal infection. This will include throat swabs and swabs of scabies/skin sores prior to the intervention and at regular intervals following the intervention.

3. Build on the existing Indigenous capacity by assisting in the development of new knowledge and skills to improve the health and well being of Indigenous communities

Outcomes

- Train at least two Aboriginal community workers in each of the participating communities in health intervention, community development strategies and research processes specific to Healthy Skin Programs
- Improve awareness of issues relating to scabies and skin sores and the link between these and other health conditions
- Develop approaches to target residual skin sores

Methods

- See below

4. Establish the feasibility of incorporating Healthy Skin Programs into existing health service delivery.

Outcomes

- Determine the costs and benefits of regional Healthy Skin Programs, and develop strategies for integration into routine service delivery
- Assess community satisfaction with the Program

Methods

- Assessment of outcomes listed under objectives 3 & 4 will be addressed through community surveys and focus group discussions conducted during the pre and post intervention period. This will assess awareness of the program, hygiene components, community infrastructure, and satisfaction with training programs.
- A separate component will monitor recurrent scabies infections or re-infections to assess whether these were related to failure to treat or treatment failure or to re-infection and how these issues could impact on sustainability of the program.
- Economic assessment of the intervention will be undertaken utilising data collected during the course of the program and based on a model to be developed in consultation with a health economist

Appendix 5
Glossary and Abbreviations

AHW	Aboriginal Health Worker
APSGN	Acute post streptococcal glomerulonephritis
ARF	Acute rheumatic fever
CRCAH	Cooperative Research Centre for Aboriginal Health
CRCATH	Cooperative Research Centre for Aboriginal and Tropical Health
DEET	Department of Employment, Education and Training
DMO	District Medical Officer
FTE	Full-time equivalent
GAA	Growth Assessment and Action program
GAS	Group A streptococcal infection
MCRI	Murdoch Childrens Research Institute
MSHR	Menzies School of Health Research
NHMRC	National Health and Medical Research Council
NT	Northern Territory
NT DHCS	Northern Territory Department of Health and Community Services
OATSIH	Office of Aboriginal and Torres Strait Islander Health
RHD	Rheumatic heart disease

References

1. Connors, C., Benger, N., Currie, B., Dowden, M., and Scarlett, M., *Top End Healthy Skin Feasibility Report*. 2001, CRCATH: Darwin.
2. Currie, B.J. and Carapetis, J.R., *Skin infections and infestations in Aboriginal communities in northern Australia.* Australas.J.Dermatol., 2000. **41**(3): p. 139-143.
3. Currie, B., Huffam, S., O'Brien, D., and Walton, S., *Ivermectin for scabies.[comment].* Lancet, 1997. **350**(9090): p. 1551.
4. Carapetis, J.R., Connors, C., Yarmirr, D., Krause, V., and Currie, B.J., *Success of a scabies control program in an Australian aboriginal community.* Pediatr.Infect.Dis.J., 1997. **16**(5): p. 494-499.
5. Breinl, A. and Holmes, M., *Medical report onthe data collected during a journey through some districts of the Northern Territory.* Bull of the Northern Territory, 1915. **15**: p. 1-8.
6. Hackett, C., *Boomerang legs and yaws in Australian Aborigines.* Trans R Soc Trop Med Hyg, 1936. **30**: p. 137-150.
7. Harris, M., Nako, D., Hopkins, T., et al., *Skin infections in Tanna, Vanuatu in 1989.* P.N.G.Med.J., 1992. **35**(2): p. 137-143.
8. Taplin, D., Lansdell, L., Allen, A.M., Rodriguez, R., and Cortes, A., *Prevalence of streptococcal pyoderma in relation to climate and hygiene.* Lancet, 1973. **1**(7802): p. 501-3.
9. Walton, S.F., Currie, B.J., and Kemp, D.J., *A DNA fingerprinting system for the ectoparasite Sarcoptes scabiei.* Molecular & Biochemical Parasitology, 1997. **85**(2): p. 187-96.
10. Carapetis, J., *An outbreak of acute post-streptococcal glomerulonephritis in an Aboriginal community.* NT Commun Dis Bull, 1994. **2**: p. 1-4.
11. Evans, C., *Acute Post Streptococcal Glomerulonephritis in the Northern Territory 1980 - 2000.* Northern Territory Disease Control Bulletin, 2001. **8**: p. 1-6.
12. Kearns, T., Evans, C., and Krause, V., *Outbreak of acute post-streptococcal glomerulonephritis in the Northern Territory - 2001.* NT Disease Control Bulletin, 2001. **8**: p. 6-14.
13. White, A.V., Hoy, W.E., and McCredie, D.A., *Childhood post-streptococcal glomerulonephritis as a risk factor for chronic renal disease in later life.* Med.J.Aust., 2001. **174**(10): p. 492-496.
14. Carapetis, J.R., Wolff, D.R., and Currie, B.J., *Acute rheumatic fever and rheumatic heart disease in the top end of Australia's Northern Territory.* Med.J.Aust., 1996. **164**(3): p. 146-149.
15. Carapetis, J.R., Currie, B.J., and Mathews, J.D., *Cumulative incidence of rheumatic fever in an endemic region: a guide to the susceptibility of the population?* Epidemiol.Infect., 2000. **124**(2): p. 239-244.
16. Carapetis, J.R., Currie, B.J., and Kaplan, E.L., *Epidemiology and prevention of group A streptococcal infections: acute respiratory tract infections, skin infections, and their sequelae at the close of the twentieth century.* Clinical Infectious Diseases, 1999. **28**(2): p. 205-10.
17. Carapetis, J.R. and Currie, B.J., *Group A streptococcus, pyoderma, and rheumatic fever.[comment].* Lancet, 1996. **347**(9010): p. 1271-2.
18. Martin, D.R., Voss, L.M., Walker, S.J., and Lennon, D., *Acute rheumatic fever in Auckland, New Zealand: spectrum of associated group A streptococci different from expected.* Pediatr.Infect.Dis.J., 1994. **13**(4): p. 264-269.
19. Skull, S.A., Krause, V., Coombs, G., Pearman, J.W., and Roberts, L.A., *Investigation of a cluster of Staphylococcus aureus invasive infection in the top end of the Northern Territory.* Aust.N.Z.J.Med., 1999. **29**(1): p. 66-72.
20. Carapetis, J.R., Walker, A.M., Hibble, M., Sriprakash, K.S., and Currie, B.J., *Clinical and epidemiological features of group A streptococcal bacteraemia in a region with hyperendemic superficial streptococcal infection.* Epidemiol.Infect., 1999. **122**(1): p. 59-65.
21. Thomas, D.P., Heller, R.F., and Hunt, J.M., *Clinical consultations in an aboriginal community-controlled health service: a comparison with general practice.* Australian & New Zealand Journal of Public Health, 1998. **22**(1): p. 86-91.
22. Porter, M.J., *An epidemiological approach to skin disease in the tropics.* Tropical Doctor, 1977. **7**: p. 59-66.
23. Taplin, D., Porcelain, S.L., Meinking, T.L., et al., *Community control of scabies: a model based on use of permethrin cream.* Lancet, 1991. **337**(8748): p. 1016-1018.
24. Centre for Disease Control, *Guidelines for community control of scabies.* 1997, NT Government Publication: Darwin.
